# The APETALA-2-Like Transcription Factor OsAP2-39 Controls Key Interactions between Abscisic Acid and Gibberellin in Rice

**DOI:** 10.1371/journal.pgen.1001098

**Published:** 2010-09-09

**Authors:** Mahmoud W. Yaish, Ashraf El-kereamy, Tong Zhu, Perrin H. Beatty, Allen G. Good, Yong-Mei Bi, Steven J. Rothstein

**Affiliations:** 1 Department of Molecular and Cellular Biology, University of Guelph, Guelph, Ontario, Canada; 2 Department of Biology, College of Science, Sultan Qaboos University, Muscat, Oman; 3 Syngenta Biotechnology, Inc., Research Triangle Park, North Carolina, United States of America; 4 Department of Biological Sciences, University of Alberta, Edmonton, Alberta, Canada; The University of North Carolina at Chapel Hill, United States of America

## Abstract

The interaction between phytohormones is an important mechanism which controls growth and developmental processes in plants. Deciphering these interactions is a crucial step in helping to develop crops with enhanced yield and resistance to environmental stresses. Controlling the expression level of *OsAP2-39* which includes an APETALA 2 (AP2) domain leads to phenotypic changes in rice. Overexpression of *OsAP2-39* leads to a reduction in yield by decreasing the biomass and the number of seeds in the transgenic rice lines. Global transcriptome analysis of the *OsAP2-39* overexpression transgenic rice revealed the upregulation of a key Abscisic Acid (ABA) biosynthetic gene *OsNCED-I* which codes for 9-cis-epoxycarotenoid dioxygenase and leads to an increase in the endogenous ABA level. In addition to *OsNCED-1*, the gene expression analysis revealed the upregulation of a gene that codes for the Elongation of Upper most Internode (EUI) protein, an enzyme that catalyzes 16α, 17-epoxidation of non-13-hydroxylated GAs, which has been shown to deactivate gibberellins (GAs) in rice. The exogenous application of GA restores the wild-type phenotype in the transgenic line and ABA application induces the expression of *EUI* and suppresses the expression of *OsAP2-39* in the wild-type line. These observations clarify the antagonistic relationship between ABA and GA and illustrate a mechanism that leads to homeostasis of these hormones. *In vivo* and *in vitro* analysis showed that the expression of both *OsNCED-1* and *EUI* are directly controlled by *OsAP2-39*. Together, these results reveal a novel mechanism for the control of the ABA/GA balance in rice which is regulated by *OsAP2-39* that in turn regulates plant growth and seed production.

## Author Summary

Hormones play an important role in controlling plant growth and development through a dynamic and complicated set of interactions. ABA and GA are well-known as antagonistic partners although the mechanism through which this occurs still needs further elucidation. In this project, we found that a transcription factor isolated from rice and coding for the AP2 domain (OsAP2-39) directly controls a key ABA biosynthetic gene (*OsNCED-1*) and also a gene that codes for a GA deactivation protein (EUI). In addition, we show that ABA induces the expression of *EUI* which in turn would lead to GA deactivation. ABA also suppresses OsAP2-39 expression which would lead to a reduction in ABA synthesis. Therefore, OsAP2-39 links the ABA production and GA deactivation processes which results in ABA/GA balance and homeostasis.

## Introduction

Plant hormones have synergistic or antagonistic effects on the physiological processes associated with growth and development. ABA and GA are hormone partners and act through a complicated network of antagonistic interactions. The coordination and interaction between phytohormone is essential to achieve normal growth and development. In Arabidopsis (*Arabidopsis thaliana*), a high endogenous level of ABA causes a reduction in the endogenous level of GA [1], and vice versa [2]. ABA generally regulates development by retarding plant growth, although there is recent evidence suggesting a growth promotion effect of ABA through reducing ethylene synthesis [3]–[5]. On the other hand, GA promotes growth and is involved in seed germination, leaf expansion, shoot and root elongation, and flowering and shoot fruit development [6]. These antagonistic hormones have a mutual biosynthesis, signalling and catabolism inhibition relationship [7].

Some components of the relationship between the GA and ABA synthesis and signalling pathways have been elucidated. For example, it has been shown that the upregulation of the ABA biosynthesis gene *XERICO* is controlled by the DELLA protein which is a negative regulator of GA response in Arabidopsis [7]. Further, GA suppression has been shown to occur through the ABA-inducible protein kinase (PKABA1) present in the aleurone layer of barley [8], [9]. In addition, the FUS3 transcription factor which specifies cotyledon identity in Arabidopsis has also been found to regulate the synthesis of ABA and GA during late embryogenesis [10], [11]. However, a number of issues regarding this relationship are still unclear [12]–[14].

Transcription factors control a variety of physiological processes through altering the expression of genes involved in metabolic pathways including hormone biosynthesis and signalling in plants. One set of these is the large APETALA2 (AP2) transcription factor family [15]. AP2 proteins are found only in plants and their unique feature is that they include the AP2 DNA-binding domain. For instant, there are 139 and 122 AP2 putative family genes in rice (*Oryza sativa* L. subsp. *japonica*) and Arabidopsis respectively [16]. The AP2 gene family plays a variety of functions throughout plant growth and development including the regulation of several developmental processes like floral organ and epidermal cell identity, and they are involved in the mechanisms used by plants to respond to various types of biotic and abiotic stresses (e.g. (Shukla et al., 2006 [17]; Tang et al., 2005 [18]). The AP2 domain specifically binds to the GCC box (the consensus DNA binding motif is AGCCGCC) which was originally identified as an ethylene response element (ERF).

The AP2 gene family has been found to control a wide range of physiological processes including through the regulation of genes involved in hormone metabolism and signalling. In Arabidopsis, the DWARF AND DELAYED-FLOWERING 1 (DDF1) [19] and the LEAFY PETIOLE (LEP) are AP2 transcription factors involved in regulating GA metabolism and signalling [20]. In addition, the JERF1 is an AP2 transcription factor which modulates the expression of an ABA biosynthesis-related gene in tobacco [21]. Ectopic expression of AP2 related genes in plants causes a variety of phenotypic changes. While overexpression of *JERF1* increases salt and cold tolerance in tobacco, overexpression of *DDF1* caused late flowering and a dwarf phenotype in Arabidopsis. These phenotypic alterations were apparently due to an alteration in the endogenous hormonal balance in the plant.

The *OsAP2-39* gene which codes for a member of AP2 family in rice was initially identified as a strong nitrogen-responsive gene and was transformed into rice. The purpose of this work was to determine its role in controlling growth and development. Expression profiling data revealed the change in the expression of a large number of genes in the transgenic lines overexpressing OsAP2-39 including some key hormone biosynthetic and catabolic genes. We found that overexpression of *OsAP2-39* increases the endogenous level of ABA in rice through a direct regulatory interaction between OsAP2-39 and a key ABA-biosynthesis gene (*OsNCED-1*). In addition, either a high level of ABA or the direct action of OsAP2-39 induces the expression of the *ELONGATION OF UPPER MOST INTERNODE I* (*EUI*) gene [22]–[24]. EUI reduces the level of the bioactive forms of GAs by epoxidizing the active GAs in rice. Therefore, overexpression of *EUI* causes a dwarf phenotype whereas mutation within this gene increases the internode length in rice. The alteration in the ABA/GA ratio due to the *OsAP2-39* overexpression leads to a pleiotropic phenotype including short stems, decreased tiller and panicle number, late flowering and a low percentage of seed filling. Consequently, transgenic plants have a much lower seed yield than does the wild-type. Therefore, *OsAP2-39* is a key participant in ABA synthesis and GA catabolism in rice and is involved in maintaining hormone homoeostasis which is crucial for normal plant growth and development.

## Results

### The Rice *OsAP2-39* Gene Sequence Analysis

**Figure 1:**
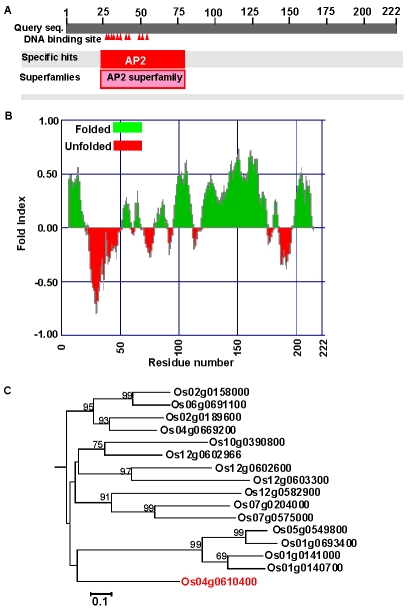
*OsAP2-39* (*Os04g52090*) has unique features among the *AP2* gene family. (A) Schematic representation of OsAP2-39 protein domain structure showing the location of the AP2 domain and the DNA-binding sites (indicated by shaded triangles). (B) A predicted secondary structure of the OsAP2-39 protein based on the hydrophobicity profile showing that it is mainly hydrophobic and codes for folded peptides. (C) Neighbour-Joining phylogenetic analysis of OsAP2-39 protein in the context of closely related AP2 proteins from rice. Tree topology with bootstrap support based on a percentage of 1000 replicates is constructed using the Clustal X and MEGA programmes. OsAP2-39 (Os04g52090) outgrouped in the tree indicating overall protein sequence dissimilarity.

The rice genome codes for 139 putative AP2 family proteins with a variety of functions and domain structures [16]. The *OsAP2-39* (Os04g0610400) cDNA was isolated by using a PCR strategy and cloned using standard protocols. The *OsAP2-39* cDNA is composed of 666 bp and the genomic sequence contains no introns. The Protein Basic Local Alignment Search Tool (BLASTP) available at the National Center for Biotechnology Information (NCBI) website (http://blast.ncbi.nlm.nih.gov/Blast.cgi) showed that *OsAP2-39* codes for a single AP2 domain present at the N-terminal part of the protein. This domain includes 11 putative DNA-binding sites (Figure 1A) implying a strong binding capacity. The OsAP2-39 protein is about 22.8 kDa with a predicted average pI of 9.62. The hydrophobicity profile indicates that OsAP2-39 domains are mostly hydrophobic and is a folded protein (Figure 1B). Sequence and phylogenetic analysis showed that OsAP2-39 is similar to other AP2 family members only within the AP2 conserved domain, but the rest of the protein sequence does not have a high level of similarity with other rice proteins (Figure 1C) or proteins from other plant species. Based on the BLASTP search results, a deduced amino acid sequence from maize (Gene Bank accession number ACG28382) has the highest degree of homology with OsAP2-39 (65% identity and 69% similarity).

**Figure 2:**
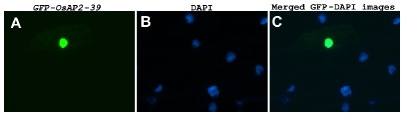
OsAP2-39 is localized in the nucleus. OsAP2-39 is fused to GFP and bombarded into onion epidermal cells. (A) GFP is in the nucleus of a transformed cell (B) Nucleus of onion epidermal cells stained with 4′-6-Diamidino-2-phenylindole (DAPI). (C) Merged image of (A) and (B).

OsAP2-39 was localized in the nucleus of the onion epidermal cells when it was fused with the C-terminal part of the GFP (Figure 2). Although *OsAP2-39* does not code for a conventional nuclear localization signal, a prediction of subcellular localization using bioinformatics tools such as LOCtree of the University of Colombia available at (http://cubic.bioc.columbia.edu/cgi-bin/var/nair/loctree/query) showed that OsAP2-39 is a nuclear protein with a 95% chance of possibility. Together the sequence analysis and the subcellular localization results of OsAP2-39 suggest that this protein is a transcription factor.

### Altering expression of *OsAP2-39* in Rice Causes Pleiotropic Phenotypes

**Figure 3:**
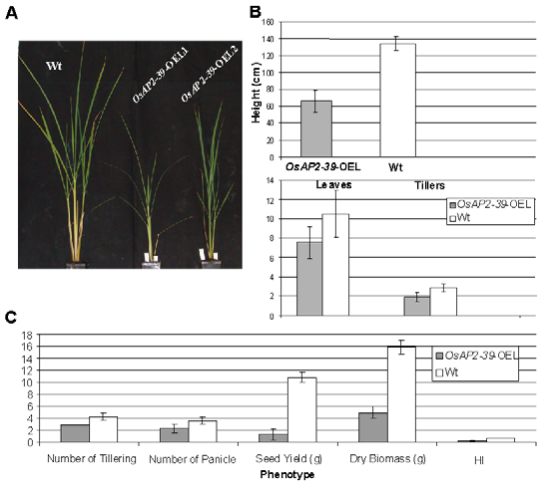
Overexpression of *OsAP2-39* causes a pleiotropic phenotype in rice and significantly reduces the yield and the Harvest Index (HI). 4-week old wild-type and transgenic rice plants demonstrates the effect of the *OsAP2-39* expression on the phenotype (A); height and number of tillers and leaves (B); and the biomass (C).

**Figure 4:**
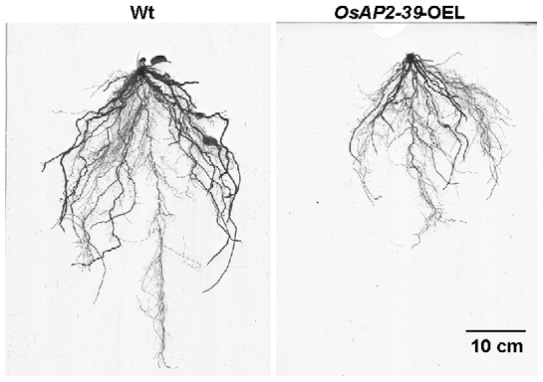
The size of roots system is significantly reduced by the *OsAP2-39* overexpression in rice. The root system of the transgenic line is about 30% smaller than the Wt.

The *OsAP2-39* cDNA was constitutively overexpressed in rice under the control of a corn ubiquitin promoter. Four independent transgenic lines were chosen for further studies using the phosphomannose isomerase (PMI) activity assay [25] as a selectable marker. These transgenic rice plants had pleiotropic phenotypes which led to overall biomass reduction (Figure 3A–3C). These included less green leaves at the 1–2-weeks old stage, shorter inter-nodes including the upper most one, fewer leaves and tillers (Figure 3B), reduction in seed yield (Figure 3C), and delays in flowering by 1 to 2 week. The transgenic plant height was reduced by 55%, tillers by 75%, and the number of the leaves by 74% comparing with the wild-type plants. Consequently the yield of the transgenic plants was less than the wild-type by about 80% (Figure 3B). The root system of the transgenic lines is also affected by *OsAP2-39* overexpression. It has about 30% less total length, surface area, average diameter, and number of tips than the wild-type (Figure 4 and Figure S1).

**Figure 5:**
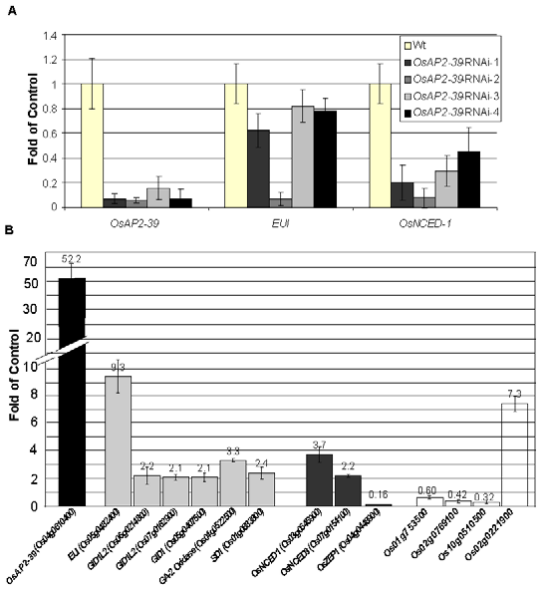
Alteration the expression of *OsAP2-39* affects the expression level of ABA and GA related genes are altered in the transgenic rice. Quantitative gene expression analysis using qRT-PCR of several genes showed variation between the wild-type and the transgenic line. (A) Knocking down the *OsAP2-39* reduces the expression of *EUI* and *OsNCED-1* in the T_0_ plants. Similar results were obtained in the T_1_ plants. (B) Overexpression of *OsAP2-39* affects various hormone related genes. Bars of the ABA, GA and auxin-related genes are shaded with grey, dark grey and white, respectively. *Actin 2* (*Os10g0510000*) was used as an internal control. Bars represent mean ±SE (n = 3).

Wild-type plants transformed with an RNAi construct designed to block the production of OsAP2-39 were made. Of the initial lines produced, 5/29 initial transformed plants showed a decrease in the level of OsAP2-39 transcript of up to 5-fold (Figure 5A). While overexpression of *OsAP2-39* leads to an increase in gene expression level of both EUI and OsNCED-1, the plants having a decreased expression of *OsAP2-39* also have a decreased level of expression of both *EUI* and *OsNCED-1* (Figure 5A). This supports the direct OsAP2-39 regulatory effect on *EUI, and OsNCED-I*. Preliminary phenotypic showed that these RNAi lines were taller and a higher tiller number. Unfortunately, the decreased expression of the OsAP2-39 gene in any of the 4 lines was not inherited in the following generation which made it impossible to confirm this phenotypic analysis.

### *OsAP2-39*-Transgenic Rice Responds to Exogenous Application of ABA and GA

Overexpression of *OsAP2-39* affects several physiological processes. This includes low germination rate and shorter internodes. Thus, overexpression of the *OsAP2-39* in rice shows a similar phenotype to that found in GA deficient mutants in plants like the rice lines harbouring mutations within various GA biosynthetic genes [26], the *gibberellin insensitive dwarf1-1 (gid-1*) [27], the *semi-dwarf-1* (*sd1*) [28], the *slender rice-1* (*slr1*) [29], and the *dwarf and gladius leaf 1* (*dgl1*) [30] mutants. In addition, the Arabidopsis mutants *gibberellin insensitive dwarf1* (*atgid1a* and *atgid1b*) [31], *sleepy1* (*sly1*) [32], [33], and *gibberellin-responsive dwarfs ga1-3* [*34*] have a similar impact on phenotype.

**Figure 6:**
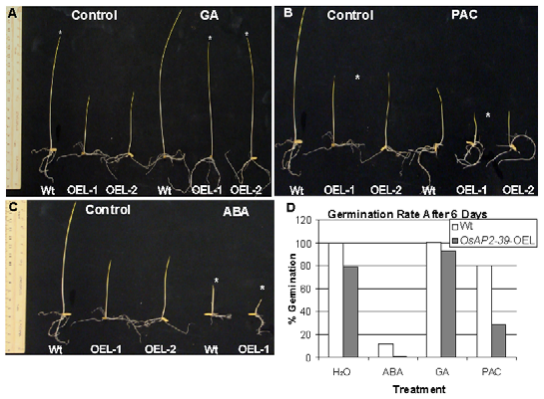
*OsAP2-39* transgenic line responds to hormonal treatment. Transgenic seedlings (*OsAP2-39-O*EL-1 and *OsAP2-39-O*EL-2) grown on 100 µM GA rescued the wild-type phenotype (A). Treatment with 10 µM ABA (B) and 10 µM PAC (C) increase the effect of the *OsAP2-39* overexpression on the phenotype. Asterisks indicate the effect of the treatment on the different genotypes. (D) The seed germination rate increased after GA treatment and was reduced when ABA and PAC were used.

Given these phenotypic similarities, GA deficiency was investigated as a reason for the phenotype caused by the overexpression of the *OsAP2-39* and GA3 was exogenously applied to the transgenic lines. In addition, the GA biosynthesis inhibitor paclobutrazol (PAC) and ABA (as a GA antagonist) were used to confirm the effect of the GA on the phenotype. Seeds from both genotypes were germinated on a filter paper saturated with different hormone solutions and the number of germinated seeds was counted after 6 days. In a separate experiment, seeds were planted in magenta boxes containing solutions of different hormone treatments and incubated in the dark for one week. The results showed that hormonal treatment can modify the phenotype in the transgenic lines (Figure 6A–6D). Treating the transgenic seeds with GA3 recovers the seedlings height (Figure 6A) and also the seed germination rate (Figure 6D). In comparison with the wild-type, treating the rice seeds with 10 µM PAC decreases the germination rate (Figure 6D) and seedlings height (Figure 6C). In addition, use of ABA delays seed germination in the transgenic lines and shows more drastic effect on the growth of transgenic lines than in the wild-type (Figure 6B). The results also showed that treating rice plants at the 4-weeks old stage with 100 µM GA for 4 weeks with a dosage of 2 times/week rescued the normal height and flowering time in the transgenic plants, although the number of tillers did not recover with this treatment(data not shown). This may reflect additional physiological processes associated with axillary bud initiation and development or could be due to inappropriate site and/or time of GA3 application.

Thus, exogenous application of the GA3 recovers the endogenous level of this hormone in the transgenic lines. This leads to a restoration of most wild-type phenotypes in the transgenic lines. On the other hand, decreasing the endogenous level of GA by the application of PAC or reducing the effect of GA by addition of ABA magnified the *OsAP2-39* effect on the transgenic line. This implies a shortage in bioactive GAs due to improper gene expression.

### Global Gene Expression Analysis Revealed Alteration in the Expression of GA Signalling and ABA Biosynthetic Genes

In order to determine the molecular events associated with *OsAP2-39* overexpression, global gene expression analysis on the transgenic rice was carried out using the Affymetrix gene chip microarrays. RNA samples were isolated from 4-week old leaves and processed for microarray analysis. Comparing with the wild-type, the gene expression analysis results showed an alteration in 409 genes in the transgenic rice lines (Table S1). The gene list includes 172 upregulated and 237 downregulated genes. Because microarray analysis may not detect every single gene whose expression is modulated in the transgenic line, the expression of additional genes involved in GA and ABA biosynthesis and signalling were tested using quantitative real time PCR (qRT-PCR) analysis. Interestingly, the results showed that the expression level of genes involved in ABA biosynthesis and GA catabolic and signalling pathways were changed due to the *OsAP2-39* overexpression (Figure 5B). This includes the upregulation of a putative *OsNCED-1 (Os03g0645900)*, and *OsNCED-3* (*Os07g0154100*) genes coding for the 9-cis-epoxycarotenoid dioxygenase which are ABA-biosynthetic enzymes [35]. The *OsNCED-1* codes for a protein with 83% identity and 90% similarity based on the Dayhoff matrix to the maize VIVIPAROUS14 (VP14) protein (Figure S2) which catalyzes the cleavage of 9-cis-epoxy-carotenoids to form C25 apo-aldehydes and xanthoxin, a precursor of ABA in higher plants. As a result, it is considered to be a key enzyme in the ABA synthesis pathway [35]. The VIVIPAROUS14 expression level is directly related with the ABA synthesis rate [35]–[37].

**Figure 7:**
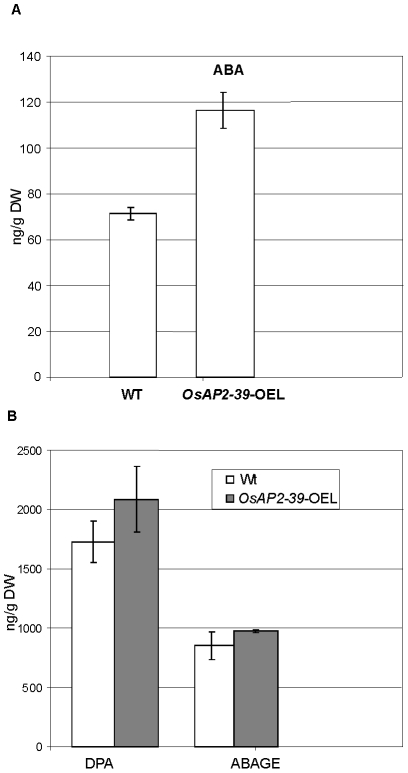
The free endogenous Abscisic Acid (ABA) and its derivatives are increased in the *OsAP2-39* transgenic rice. (A) ABA content in the transgenic lines is about 2-fold higher than the wild-type. (B) The ABA derivatives Dihydrophaseic acid (DPA) and Abscisic acid glucose ester (ABAGE) levels are also increased in the rice transgenic line. Hormones were extracted from two pools of 8 leaves taken form two groups of transgenic lines and the Wt. Bars represent mean ±SE (n = 2).

Consistent with this observation, the active endogenous ABA level of the *OsAP2-39* transgenic rice lines was found to be 2-fold higher than the wild-type level (Figure 7A). In addition, the ABA derivative compounds such as Dihydrophaseic acid (DPA) and Abscisic acid glucose ester (ABAGE) levels are also increased in the rice transgenic line (Figure 7B). In addition to the *OsNCED* genes, the rice *Zeaxanthin epoxiydase* (*OsZEP-1*) (*Os04g0448900*) was downregulated in the transgenic line. These genes are involved in the ABA biosynthesis pathway. Free ABA is deactivated by oxidation to phaseic acid and by the formation of glucose conjugates. Induction of ABA oxidation may result from a feed back inhibition loop interaction due to the excessive level of the endogenous ABA in the transgenic line. Knockouts of the *OsZEP-1* caused dwarf rice mutants [38] and the relative abundance of *AB2*, which codes for a Zeaxanthin epoxiydase in tobacco (*Nicotiana plumbaginifolia*) is reduced due to the increase level of ABA [39]. These previous observations are consistent with the phenotype and the ABA level obtained in the *OsAP2-39* overexpressed lines.

The gene expression analysis also showed the upregulation of the *ELONGATED UPPERMOST INTERNODE* (*EUI*) (*Os05g0482400*) gene which encodes for a cytochrome P450 monooxygenase, an enzyme which deactivates gibberellin through an epoxidation reaction [22]–[24]. GA deactivation can occur through other mechanisms. For example, in Arabidopsis GAs are deactivated through GA 2-Oxidase including AtGA2ox7 and AtGA2ox8 [40] and GA oxidase-6 (AtGAox6) [41]. The microarray and qRT-PCR data showed that a putative gibberellin 2-beta-oxidase7 (Os04g0522500) is upregulated in the *OsAP2-39* transgenic line. In addition, the microarray and qRT-PCR data showed the upregulation of 3 gibberellin receptor proteins: *OsGID1* (*Os07g0162700*) and GID1L2 (*Os06g0214800*, *Os07g0162900*) (Figure 5B).

This result indicates a regulatory role of *OsAP2-39* on GA activity in the transgenic line. Exogenous application of GA3 recovers the wild-type phenotype and application of the GA inhibitor PAC magnifies the effect of OsAP2-39 on the phenotype indicating a low endogenous content of active GA in the transgenic line. Analysis of the endogenous level of GAs revealed alterations in the hyroxylated GAs in the *OsAP2-39* overexpression lines (Table 1). However, non-13-hydroxylated GAs was under the detectable limits. The non-13-hydroxylated GAs are supposed to be the EUI substrates in rice and similar results were previously obtained when the endogenous non-13-hydroxylated GA levels in the *EUI* overexpressed line were measured even after an exogenous treatment with GA3 [24].

**Table 1:** Endogenous GAs level in rice (ng/g dry weight).

	GA1	GA3	GA4	GA8	GA19	GA20*	GA29*	GA34	GA53*	GA51
Wt	0.95	0.63	N.D.	0.38	9.28	2.21	0.27	N.D.	10.69	N.D.
***OsAP2-39*** **-OEL-1**	1.16	2.25	N.D.	0.47	9.03	3.09	0.18	N.D.	8.08	N.D.
***OsAP2-39*** **-OEL-2**	0.7	0.8	N.D.	0.38	9.3	2.86	0.16	N.D.	7.95	N.D.

Two *OsAP2-39* expression lines (OEL) and one Wt lines has been tested. The results represent the mean of three readings.

*: Showed consistent changes.

### *EUI* Is Induced by Endogenous and Exogenous ABA

**Figure 8:**
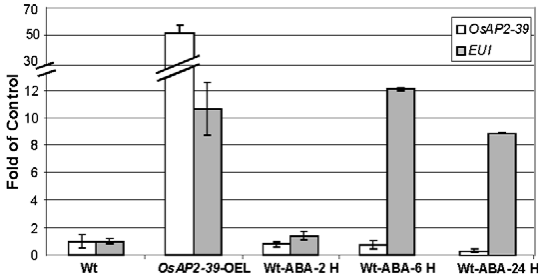
Exogenous application of ABA induced *EUI* in the wild-type rice leaves. A quantitative gene expression study using the qRT-PCR analysis of *EUI* after 2 hours (2 H), 6 hours (6 H) and 24 hours (24 H) of 10 µM ABA application showing that *EUI* is highly upregulated in rice leaves when treated with ABA after 6 hours. Actin 2 was used as an internal control. Bars represent mean ±SE (n = 3).

Gene expression analysis revealed the upregulation of *EUI* in the *OsAP2-39* transgenic rice. *EUI* encodes an enzyme that deactivates GA by catalyzing 16α, 17-epoxidation of non-13-hydroxylated GAs. At the same time, the transgenic lines have a higher endogenous ABA level than the wild-type. Since the overexpression of *EUI* is associated with a high level of ABA in the transgenic lines, the physiological relationship which links *EUI* with ABA was tested. Wild-type rice plants were sprayed with 10 µM ABA and the expression of *EUI* in leaves after 1, 6, 24 hours of ABA application was measured using qRT-PCR. The qRT-PCR results revealed that ABA induces *EUI* with a maximum level of expression after 6 hours of ABA treatment (Figure 8). Consistent with this result, sequence analysis of the *EUI* promoter using the Plant *Cis*-acting Regulatory DNA Elements (PLACE, http://www.dna.affrc.go.jp/PLACE/signalscan.html) showed the presence of one ABA Response Element (ABR) motif (CACGTG) in the *EUI* promoter at −2355 bp from the ATG start codon. These results show that a high endogenous ABA level is responsible at least in part for the *EUI* induction in the transgenic rice line and would explain how ABA is able to reduce bioactive GAs. After exogenous ABA treatment, *OsAP2-39* is down regulated demonstrating a feed back mechanism leading to a reduction in the endogenous production of ABA (Figure 8).

### Recombinant *OsAP2-39* Protein Binds to the GCC-Box *In Vitro* and Activates the ABA-Biosynthesis Gene *OsNCED-1* and GA Catabolic Gene *EUI In Vivo*

**Figure 9:**
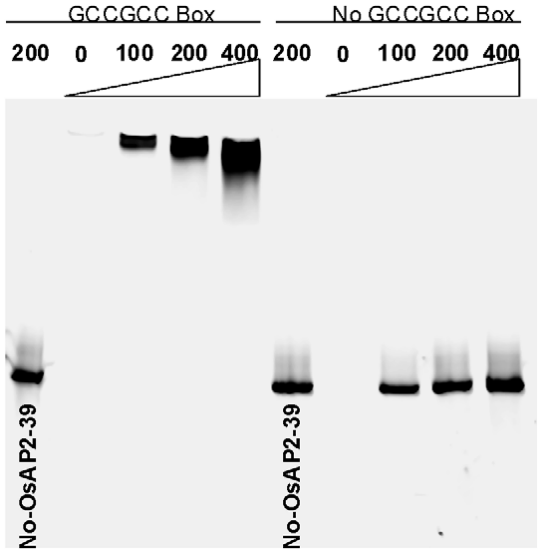
The recombinant OsAP2-39 protein specifically binds to the GCC-box *in vitro*. Electrophoretic Mobility Shift Assay (EMSA) showing the binding of OsAP2-39 Protein to 100, 200 and 400 ng of DNA containing one copy of the GCC-box. The DNA-binding capacity of the protein was not detected in a mutated DNA sequence missing the GCC-box.

The gene expression analysis revealed that *OsNCED-1* and *EUI* are upregulated in the *OsAP2-39* transgenic lines. In order to investigate the mechanism of *OsNCED-1* and *EUI* upregulation, the DNA sequences corresponding to the promoters of the both genes was analyzed using the PLACE software. The results showed that the *OsNCED-1* promoter has 3 GCC sequence motifs located at 610, 742, and 1027 bp from the first ATG codon of the cDNA. Likewise, the sequence analysis showed that the *EUI* promoter has one GCC box located at 2488 bp from the first ATG codon of the cDNA. This motif is usually considered to be a binding box for AP2 transcription factors and therefore is a potential binding site for OsAP2-39. To check the possibility that the OsAP2-39 protein binds to the GCC-box *in vitro*, recombinant OsAP2-39 protein was produced in *Escherichia coli* (*E. coli*) and used for Electrophoretic Mobility Shift Assays (EMSA). The results demonstrate that OsAP2-39 strongly binds to the *OsNCED-1* promoter sequence containing the GCC box motif. Substitution of the GCC box with poly adenine and thiamine sequence (5′-ATATAT-3′) inhibited the OsAP2-39-binding capacity to the DNA sequence (Figure 9). This result indicates an *in vitro* binding specificity of the OsAP2-39 to the GCC DNA motif.

**Figure 10:**
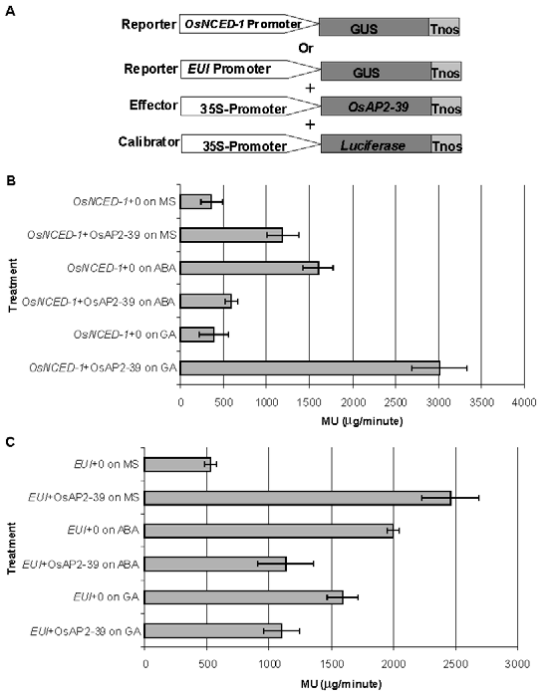
The OsAP2-39 transcription factor directly activates *OsNCED-1* and *EUI* and its activity is affected by the environmental hormonal conditions. Transcription activation of OsAP2-39 using GUS based transient assay and 4-methylumbelliferyl β-D-glucuronide (MUG) as the substrate. (A) Illustration of the reporters, the effector and the loading calibrator constructs (not drawn to scale). Tobacco leaves were bombarded with the construct containing the *OsNCED-1* (B) or the *EUI* promoter (C) fused to the GUS gene and then leaves were incubated on MS media supplemented with water (0), 10 µM ABA or 100 µM GA. The 4-methyl umbelliferone (MU) was used to generate the standard curve. Bars represent mean ±SE. (n = 8).

In order to investigate a direct relationship between the OsAP2-39 protein and *OsNCED-1* and *EUI* gene expression, a transcription activation assay using a transient gene expression strategy was carried out using β-glucorinidase (GUS) as a reporter protein. A fusion construct was made either between the promoter region of *OsNCED-1* or *EUI* and the GUS cDNA. In a separate construct, *OsAP2-39* cDNA was cloned under the control of the 35S constitutive promoter and used as a transcription activator in the experiment. The pJD312 containing the firefly (*Photinus pyralis*) luciferase cDNA driven by the CaMV 35S promoter was used as the loading DNA control and the luciferase activity used to normalize the GUS activity in every sample (Figure 10 A). DNA from the three vectors was co-transformed into the tobacco leaves using the particle bombardment method, with the empty vectors used as negative controls. Tobacco leaves were incubated 40 hours on Murashige and Skoog basal salt mixture (MS) solid media supplemented with ABA or GA at room temperature before protein from the leaves was isolated and used in the quantitative GUS and luciferase assays. The results demonstrated that OsAP2-39 slightly activates the expression of *OsNCED-1* in tobacco epidermal cells when it is incubated on MS hormone-free medium. However, when the MS was supplemented with 100 µM GA, the OsAP2-39 was able to induce *OsNCED-1* by almost 8 fold compared with the control experiment (Figure 10B). Incubation of the bombarded tobacco leaves on MS media containing 10 µM ABA induces the expression of OsNCED-1 in the absence of the OsAP2-39 activator protein. However, in the presence of the OsAP2-39, the expression of *OsNCED-1* was reduced to 1/3 when compared with the control experiment. Interestingly, the results also showed that OsAP2-39 is able to highly activate the *EUI* promoter in the tobacco cells if incubated on hormone-free MS medium. In addition, the results demonstrated that *EUI* is induced by ABA and this induction was reduced in the presence of OsAP2-39 (Figure 10B).

Together these results indicate that OsAP2-39 directly regulates the expression of both *OsNCED-1* and *EUI* and that this regulation is modulated by other factors induced by ABA and GA. OsAP2-39 was found to be more active in upregulating the *OsNCED-1* gene in a high GA environment, which would lead to an increase in the ABA content.

### Expression Pattern of the *OsAP2-39*

**Figure 11:**
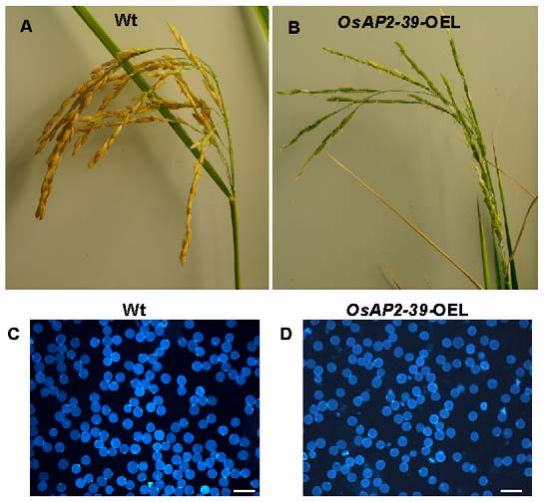
The overexpression of *OsAP2-39* in the flowers caused a reduction in the rice yield. A spikelet from Wt (A) and the *OsAP2-39* overexpressed line (*OsAP2-39-*OEL) (B) shows a reduction in the number of filled seeds in the transgenic lines. Both spikelets are dissected from plants of the same age although the seeds of the Wt mature 2 weeks before the transgenic lines. Pollen grains isolated from rice Wt flowers (C) and the *OsAP2-39* overexpressed line (D) and tested under the microscope. Bar = 2 mm.

Rice lines transformed with the *OsAP2-39* gene have fewer filled seeds in the spiklets (11A–B). Therefore it was of interest to determine whether the *OsAP2-39* gene affects the pollination and fertilization processes in the flower. This was analyzed by investigating pollen grain morphology and viability. Compared to the wild-type, the results showed that *OsAP2-39* overexpressing plants produced slightly smaller pollen grains and had a higher percentage with an irregular shape (Figure 11C–11D). Similar observations were previously obtained when rice was treated with both cold and ABA [42]. In addition, a low active GA level due to *EUI* overexpression also leads to inhibited seed production in the transgenic lines [24]. This fact highlights the contribution of ABA and GA in this phenotype.

Our findings that *OsAP2-39* affects pollen grain morphology is consistent with the rice microarray data available through the public GENEVESTIGATOR database [43] which demonstrates that *OsAP2-39* is highly expressed in rice anthers. Microarray and qRT-PCR data presented in this work showed that *OsAP2-39* is expressed in the root at the early booting stage (Figure S3), when the ABA level is elevated in some grass plants such as barley [44].

In order to confirm the site of *OsNUE39* expression in the plant tissue, the *OsAP2-39* promoter was fused to the *GUS* reporter gene and transformed into Arabidopsis wild-type plants (Figure S4). Histochemical staining of GUS showed that *OsNUE39* is predominantly expressed in the roots of the seedling (Figure S4A), roots of adult plants (Figure S4B); and in the pollen grains (Figure 4SD–4SF). This result is consistent with the microarray and RT-PCR data obtained from rice tissues.

### The Effect of *OsAP2-39* on Dehydration Tolerance

The plant hormone ABA regulates tolerance to environmental stresses such as drought and cold. In order to study the influence of high ABA on stress tolerance, the transgenic *OsAP2-39* and wild-type plants were treated under cold and water stress conditions. While cold treatment did not show any specific effect on the transgenic lines, leaves of the transgenic lines are more susceptible to dehydration conditions than the wild-type.

**Figure 12:**
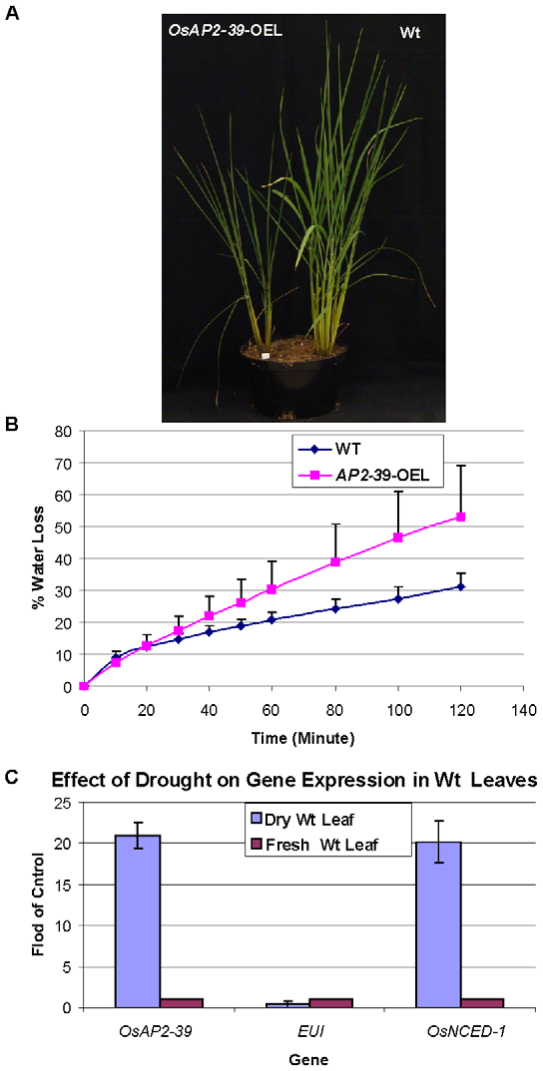
The *OsAP2-39* does not increase drought tolerance in rice. Wt and transgenic rice plants were grown in the same pot (A). When grown together, Wt was more tolerant to drought than the transgenic line. (B) Water loss assay showing that leaves from the transgenic line loses water faster than the Wt. (C) Gene expression analysis of *OsAP2-39*, *OsNCED-1* and *EUI* from Wt rice leaf tissue harvested from plants grown under drought conditions demonstrating that *OsAP2-39* and *OsNCED*-1 are upregulated, while *EUI* is down regulated under these conditions. Bars represent mean ±SE.

Drought tolerance experiments were carried out following the procedures described earlier by Yu et al. 2008 [45]. When plants of the two genotypes were grown in two different pots under water deprivation, wild-type plant dried faster than the *OsAP2-39* overexpression line probably because of their large biomass which normally reflects a higher transpiration rate (Figure S5). When the two genotypes were grown in the same pot under water deprivation, the wild-type was able to grow for a longer time than the *OsAP2-39* overexpression line likely due to their larger root system (Figure 12A). Therefore it was difficult to reach any definitive conclusions from these two experiments. In order to better clarify this issue, an excised leaf water loss assay was done and the results showed that the transgenic *OsAP2-39* lost water faster than does the wild-type (Figure 12B) indicating that *OsAP2-39* has a lower leaf dehydration tolerance than the wild-type.

It has been shown that dehydration induces ABA synthesis in plants. In order to determine the effect of dehydration on *OsAP2-39* and *OsNCED-1* expression, RNA was extracted from rice leaves dehydrated for 2 h and tested using qRT-PCR. The results show that *OsAP2-39* and *OsNCED-1* are highly induced by dehydration (Figure 12C) and this in turn would lead to an increase in ABA synthesis in that tissue. However, given that the overexpressing OsAP2-39 lines are not more resistant to dehydration implies that the increased production of ABA is not sufficient for drought tolerance. Despite the fact that a high ABA level is normally associated with stomatal closure and therefore drought tolerance, it is possible that the guard cells in the OsAP2-39 lines did not also have a higher ABA content.

## Discussion

**Figure 13:**
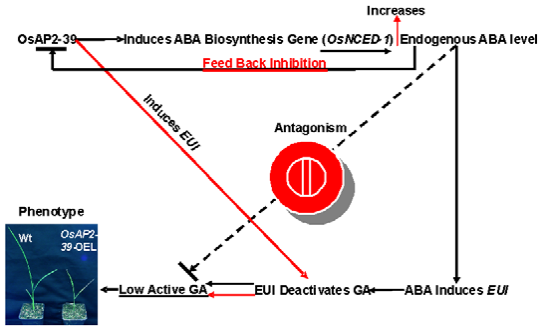
A schematic diagram of the involvement of OsAP2-39 in the ABA and GA antagonism and homeostasis. The *OsAP2-39* controls the level of bioactive GA through two different pathways. First is an indirect pathway (presented in black continuous arrows) in which the OsAP2-39 activates the ABA biosynthesis gene (*OsNCED-1*) which increases the endogenous ABA level in rice. A high ABA level induces *EUI* expression which in turn deactivates GAs. Second is a direct pathway (presented in red continuous arrows) in which the OsAP2-39 transcription factor directly upregulates *EUI*. A high ABA content down regulates *OsAP2-39* leading to ABA homeostasis. Both mechanisms lead to a reduction in bioactive GAs and consequently the appearance of the abnormal phenotypes seen in the *OsAP2-39* overexpressing lines (*OsAP2-39-*OEL).

Phytohormones regulate plant growth and development through a complex set of interactions. ABA and GA represent an example of a multidimensional and antagonistic relationship which has been studied over the last few decades. However, many important aspects of this relationship remain undiscovered. Here we demonstrate that a transcription factor containing the AP2 DNA–binding domain (OsAP2-39) regulates ABA and GA crosstalk and homeostasis in rice. A hypothetical mechanism by which *OsAP2-39* controls active ABA and GA levels is shown in Figure 13. Overexpression of this transcription factor leads to an increase in the ABA content, which in turn reduces plant biomass and delays development. The mechanism by which OsAP2-39 controls the active ABA and GA is complicated and affected by the hormonal status in the tissue. OsAP2-39 slightly increases the expression of the *OsNCED-1*, which is an ABA biosynthetic gene (Figure 10B), in a hormone free environment. However, a high GA content leads to the upregulation of its expression by about 8 fold (Figure 10B). As a result, high GA in turn activates the expression of *OsNCED-1* which has been shown to be directly proportional to the ABA content in rice as observed in this study and also in other plant species where *OsNCED-1* orthologues showed the same effect [46], [47].

OsAP2-39 can induce *EUI* expression which in turn has previously been shown to reduce the bioactive forms of GAs [22]–[24]. In this study we found that this can occur through two different pathways. The first is an increased ABA content and the second is through the direct regulation of *OsAP2-39*. As a result of these two pathways, the bioactive form of GA decreases and of ABA increases. In order to retain homeostasis, high ABA content inhibits *OsAP2-39* gene expression. This set of mechanisms represents one of the pathways in which GA and ABA interact and communicate. In support of this hypothesis, the transactivation results in tobacco cells are consistent with the expression behaviour of the *OsNCED-1* and *EUI* in rice leaves when they are subjected to exogenous application of ABA and GA. The increase in the active ABA level might be due to the inhibition of the ABA catabolism pathway. However the microarray data from the *OsAP2-39* overexpression line did not show any expression alteration in the genes involved in this process.

Overexpression of the *OsAP2-39* leads to changes in the expression pattern of a large number of genes (Table S1). Some of these are due to the direct action of this transcription factor. For example, *OsNCED-1* and *EUI* are both directly regulated by OsAP2-39 (Figure 10). This in turn would lead to an alteration in hormonal balance and change the level of expression of many other genes which together causes the pleiotropic phenotype present in these lines. This includes shorter internodes, a delay in flowering, smaller root mass, decreased tiller number and a lower seed yield. All of these phenotypes can be explained by the increase in ABA content and decrease in active GA except for the decreased tiller number. Information obtained from the global gene expression analysis provides a possible mechanism for this decrease in tiller number. These lines had a decreased level of the putative auxin-responsive proteins (Os02g0769100, Os10g0510500 and Os01g0753500) and also the upregulation of a gene (*Os02g0221900*) which encodes a protein with similarity to MORE AXILLARY BRANCHES-1 (MAX1), a protein that regulates the rate of polar auxin transport in Arabidopsis [48]–[51]. Mutations within the *MAX1* gene in Arabidopsis increase the number of axillary branches; and overexpression of this gene causes less axillary branches [49]. Therefore, one can hypothesize a similar situation for the *OsAP2-39* overexpressing lines and a possible role for auxin transportation and signaling in the tillering phenotype akin to that seen in Arabidopsis for the auxiliary branching trait. Certainly, the idea that the axillary branching mechanisms in both rice and Arabidopsis are controlled by a common pathway is a reasonable one. The *MONOCULM 1* (*MOC1*) gene has been characterized and was found to control the rice axillary branches [52]. However, the expression level of this gene was not affected in the *OsAP2-39* overexpressing lines indicating a different mechanism for the alteration in axillary branching in these transgenic lines. Given the notion that *OsAP2-39* is not found in Arabidopsis, and so far only in the rice and maize sequence databases, it would not be surprising if this gene controls ABA and GA levels and axillary branching via a novel mechanism.

A high ABA content is frequently associated with cold and drought tolerance in plants. When the transgenic and wild-type lines were grown together under water deprivation, the transgenic lines were more susceptible to water stress. These observations were confirmed when transgenic excised leaves lost water faster than did wild-type leaves.

The constitutive overexpression of *OsAP2-39* leads to changes in pollen grain morphology. This may explain the low seed yield in the transgenic lines. In fact, treatment of rice panicles with ABA induces pollen sterility and subsequently causes a significant reduction in grain number due to premature spikelet abortion [42], [53]. Therefore, the fact that the transgenic lines overexpressing *OsAP2-39* have a higher ABA content is consistent with the phenotype obtained where seed yield was reduced by about 80%. A low seed yield has been also observed when *EUI* gene was overexpressed in rice plants [24]. This result further supports the relationship between ABA synthesis and GA catabolism in rice.

In conclusion, overexpression of the *OsAP2-39* gene leads to a range of altered phenotypes that reduce biomass and seed yield including shorter internodes, delayed flowering, and lower tiller number. There are a large number of genes whose expression is altered in these lines. Some of these including key control genes regulating active ABA and GA levels are regulated directly by this transcription factor while many others no doubt are altered by changes in hormone levels. This work demonstrates for the first time a relationship between ABA biosynthesis and GA catabolic genes in rice. This relationship links the production of ABA and the inhibition of active GA and thus, provides a direct link in the antagonistic interactions between these hormones.

## Materials and Methods

### Plant Growth Conditions and Hormone Treatment

Rice (*Oryza sativa* L. Kaybonnet) was grown in a growth chamber with a 16 h light cycle, at 29°C during the day and 23°C during the night. Humidity was maintained at 70%. Plants were grown in pots containing 75% vermiculite and 25% peat moss and watered weekly with a nutrient solution [54]. Four-week old rice plants were treated with 100 µM GA or 10 µM ABA twice a week constitutively for four weeks.

### *OsAP2-39* Transgenic Rice

The construct for *OsAP2-39* overexpressing was made using the maize ubiquitin promoter. *OsAP2-39* transgenic rice lines were generated using *Agrobacterium*-mediated transformation method and positively transgenic plants were selected using Phosphomannose isomerase (PMI).

### *OsAP2-39* Interference RNA (RNAi)

DNA sequence of low similarity to other rice genes located at the 3′ end (314–664 bp) of *OsAP2-39* was amplified by PCR using the following primers: AP2SiRNAF (5′- CACCTCGTCAGCCCGACCAGCAGCACG-3′) and AP2SiRNAR (5′- CTCCTCGATCGGCGGCGGCAG-3′), cloned into TOPO pENTER vector (Invitrogene), and the inverted DNA sequences separated by a GUS intron sequence were generated by site specific recombination method in the pANDA binary vector [55] down stream the maize ubiquitin promoter using the Gateway LR Clonase Enzyme Mix (Invitrogene). Transgenic rice lines were obtained using *Agrobacterium*-mediated transformation and the positive lines were selected according to Miki et al. [56].

### Root Analysis

Roots of the wild type and transgenic plants were collected from three weeks old plants growing in turface supplemented with a full slow realize fertilizer (1 g/plant). Roots were scanned and analyzed using the WinRHIZO software (v. 5.0, Regent Instruments, Inc., Quebec, QC, Canada).

### Endogenous ABA and GA Analysis

Five leaves from two OsAP2-39 transgenic rice lines and also from wild-type were pooled, freeze dried and the ABA contents were quantified at the hormone profile laboratory in the National Research Council, Plant Biotechnology Institute (NRC-PBI) Saskatoon, Canada and the method described by Chiwocha et al. [57]. Endogenous GA analysis using GC-MS was carried out using a MAT95XP mass spectrometer according to the previously published protocol [58].

### Sequence Analysis

The BLAST search program (http://www.ncbi.nlm.nih.gov/BLAST/) was used to look for protein sequences homologous to OsAP2-39 and map the protein domains. Rice sequences with highest BLAST homology score were downloaded and used for the phylogenetic analysis using the Molecular Evolutionary Genetics Analysis (MEGA4) software [59]. The neighbor-joining tree was generated with the Poisson correction method using the same software. Bootstrap replication (1000 replications) was used for a statistical support for the nodes in the phylogenetic tree.

### Subcellular Localization of *OsAP2-39*

The *OSAP2-39* cDNA sequence was cloned in frame with the GFP protein under the control of the 35S promoter. The *OSAP2-39* cDNA sequence was amplified by PCR using the following primer pair: APHindIII-ECoRIF: (5′-CCCAAGCTTATGGCTCCCAGGAACGC-3′) and APNdeI-ECoRR: (5′-CCGGAATTCCTACGCCTCCTCGATCG-3′). After digestion with *Hind*III and *Eco*RI, the PCR products were cloned into pRLT2-GFP plasmid (kindly provided from Dr. Robert Mullen, University of Guelph), amplified in *E. coli*, and transformed by particle bombardment into onion epidermal cells.

### Histochemical staining for GUS activity

A DNA sequence spanning the 2019 bp of the *OsNUE39* promoter was amplified by PCR using the following primer pairs: promoterAp2F (5′-CGCGGATCCAATCTTGCTAAAATTTTGGCAAAG-3′) and promoterAp2R (5′-CATGCCATGGGTCCGTTCTTGTTCGGGTCG-3′) and cloned into the *Bam*HI and *Nco*I sites upstream the GUS reporter gene of the pCAMBIA 3301 vector (CAMBIA institute, Australia). Then the construct was stably transformed into the Wt Arabidopsis Col and various tissues of the transformed lines were assayed for GUS activity using the standard protocol.

### Recombinant Protein Production and EMSA

The recombinant full-length OsAP2-39 protein was expressed and purified using the Intein Mediated Purification with an Affinity Chitin-binding Tag system (IMPACT) (New England Biolabs, ww.neb.com) according to the manufacturers' instructions. The *OsAP2-39* cDNA sequence was amplified by PCR using the following primer pair: APNdeI-ECoRF: (5′-GGAATTCCATATGGCTCCCAGGAACGCC-3′) and APNdeI-ECoRR: (5′-CCGGAATTCCTACGCCTCCTCGATCG-3′). The PCR product and the pTYB12 plasmid (New England Biolabs) were digested with *Eco*RI and *Nde*I. After ligation, the construct was amplified in *E. coli* cells DH10B and transformed to the *E. coli* expression host strain ER2566 cells. Electrophoretic Mobility Shift Assay (EMSA) was carried out using the recombinant OsAP2-39 protein and the DNA products obtained using the PCR. The GGCGGC-box containing DNA sequence was amplified from the OsNCED-1 promoter using the following pair of primers: PMSA2bF: (5′-AATGTCTGCGGCGCCGGCGGC-3′) and PMSA2bR: (5′-AGTGTTCTGTTCCCCCGGGGAGATAAACCC-3′). As a negative control, DNA in the EMSE reaction, the GCCGCC box motif sequence within the promoter was replaced by (5′-ATATAT-3′) using the site directed mutagenesis PCR and the following primer pair: PEMSA4F: (5′-AATGTCTGCGGCGCTATATACTGCGGTGTTTGTT-3′) and PEMSA4R: (5′-AGTGTTCTGTTCCCCCGGGGAGATAAACCC-3′). The EMSA assay was carried out using the EMSA kit (E33075) from Invitrogen (Invitrogen, www.Invitrogen.com). After purifying the PCR product, a serial dilution of DNA (0, 100, 200, 300, and 400 ng) were mixed and incubated with 30 ng of the purified OsAP2-39 recombinant protein according to the manufacturers' instructions. The DNA/protein complex samples were loaded into a Ready Gel TBE, gradient 4–20% polyacrelamide native gel (Bio-rad Laboratories, www.bio-rad.com) at 200 V for 45 minutes. The DNA in gel was stained using the SYBR Green provided in the same kit.

### Quantitative GUS Activation Analysis

As potential targets to OsAP2-39 transcription factor, DNA sequences corresponding to the *OsNCED-1* and *EUI* promoters were cloned in an intron containing GUS reporter plasmid. The DNA sequence (1 kb upstream the ATG start codon of the cDNA) of *OsNCED-1* was amplified from the rice genomic DNA using the following promoter pair: OSNCEDF: (5′-CAATAACTGCAGGACGAGACCCTTTGCCG-3′) and OSNCED-1R: (5′-AGGGAATTCTCGATCGCACAACAATCTGAGC-3′). The DNA sequence corresponding to the *EUI* promoter (2498 bp upstream the ATG start codon of the cDNA) using the following primer pair: EUallF1: (5′-CTTTGCATTTGCCGCCGTGTT-3′) and EUallR1: (5′-GGCAGCCTACTCTCTCTTTCCCCG-3′). After digestion with *Pst*I and *Eco*RI, the PCR products were cloned into the pCAMBIA1391Z vector (CAMBIA institute, Australia, www.cambia.au). *OsAP2-39* induced by the 35S promoter in the pEGAD plasmid was used as an activator protein in the co-transformation transient expression analysis. To normalize the GUS activity values, firefly (*Photinus pyralis*) luciferase driven by 35S promoter in the pJD312 plasmid (kindly donated from Dr. Virginia Walbot, Stanford University) was used. Equal amounts of DNA from the different plasmids constructs was transformed by the particle bombardment to 4-weeks old tobacco (*Nicotiana plumbaginifolia*) leaves. After incubation for 40 hours at room temperature in the dark, the total protein was extracted from each sample and GUS and luciferase activities were measured.

GUS activity was determined by measuring cleavage of β-glucuronidase substrate 4-methylumbelliferyl β-D-glucuronide (MUG) [60]. Luciferase activity was measured using the Luciferase Assay System kit (Cat. E1500) (Promega, www.promega.com) following the manufacturers' instructions. Empty vectors were used as negative controls in this experiment.

### Microarray Hybridization and Data Analysis

Double-stranded cDNAs was synthesized from 5 µg of total RNA from each sample. Labeled complementary RNA, synthesized from the cDNA was hybridized to Affymetrix rice whole genome array (Cat. Number: 900601). The hybridization signal of the arrays was obtained by the GeneChip scanner 3000 and quantified by MAS 5.0 (Affymetrix). The probe set measurement was summarized as a value of weighted average of all probes in a set, subtracting bottom 5% of average intensity of the entire array using a custom algorithm. The overall intensity of all probe sets of each array was further scaled to a target intensity of 100 to enable direct comparison. Data was analyzed using GeneSpring software (Agilent, CA, USA). The data was normalized with a default setting of the program, followed by gene filtering which required that each gene must have either a ‘P’ or ‘M’ flag in the three replicate samples. Genes with 2-fold change were identified first, and then ANOVA was used to identify significant genes (Welch t-test p-value cutoff at 0.05).

### qRT-PCR

For each genotype, leaf tissues from at least six plants were collected and pooled. The samples from 3 different pools were homogenized in liquid nitrogen prior to RNA isolation suing Tripure reagent (Roche, http://www.roche-applied-science.com). cDNA was synthesized using the qScript cDNA Supermix (Quanta Biosciences, http://www.quantabio.com/). The qRT-PCR reactions were carried out using the SYBR Green PCR Master Mix (Applied Biosystems, www3.appliedbiosystems.com) and the primers mentioned in Table S2.

## Supporting Information

Figure S1 A comparative analysis between the roots systems in wild-type (Wt) and *OsAP2-39* transgenic line (*OsAP2-39OEL*). Bars represent mean ±SE (n = 4).
(0.06 MB TIF)

Figure S2 Sequence alignment of the deduced amino acid sequences of the maize *VP14* and the rice *OsNCED-1*.
(2.52 MB TIF)

Figure S3 The *OsAP2-39-1* expression level measured using microarray technique in various rice plant tissues throughout the plant growth and development. Data present the absolute value of gene expression.
(0.61 MB TIF)

Figure S4 *OsNUE39* is expressed in the pollen grains and root of Arabidopsis. The expression pattern of *OsNUE39* determined by GUS staining of *OsNUE39* promoter fused to GUS in Arabidopsis Wt. *OsNUE39* is localized in the root and hypocotyls of the seedlings (A) and roots of the mature plants (B) and also in the flowers (C). (D) A closer look showing the GUS stain in the anthers and on the stigma during pollination. (E) GUS stains in the anther sac. (F) Dissected pollen stained with GUS. Bar = 2 mm.
(1.41 MB TIF)

Figure S5 *OsAP2-39-1* overexpression lines (*OsAP2-39*-OEL) dried slower than the wild-type rice pants due to low water consumption.
(5.04 MB TIF)

Table S1 Genes expressed differentially in the *OsAP2-39* transgenic rice leaves.
(0.74 MB DOC)

Table S2 Primers used in the qRT-PCR.
(0.05 MB DOC)
