# Supplementary figures and images for: The APETALA-2-Like Transcription Factor OsAP2-39 Controls Key Interactions between Abscisic Acid and Gibberellin in Rice
Source: PLoS Genet. 2010 Sep 9;6(9):e1001098. doi: 10.1371/journal.pgen.1001098 (PMC2936520; doi:10.1371/journal.pgen.1001098)

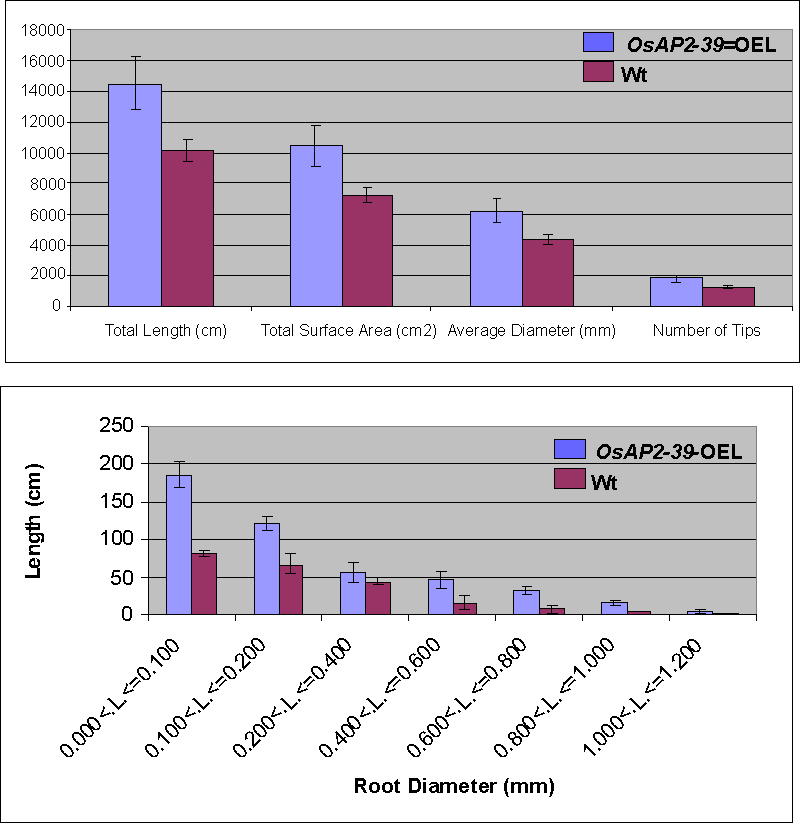

Supplement: Figure S1 — A comparative analysis between the roots systems in wild-type (Wt) and OsAP2-39 transgenic line (OsAP2-39OEL). Bars represent mean ±SE (n = 4). (0.06 MB TIF) [file pgen.1001098.s001.tif]

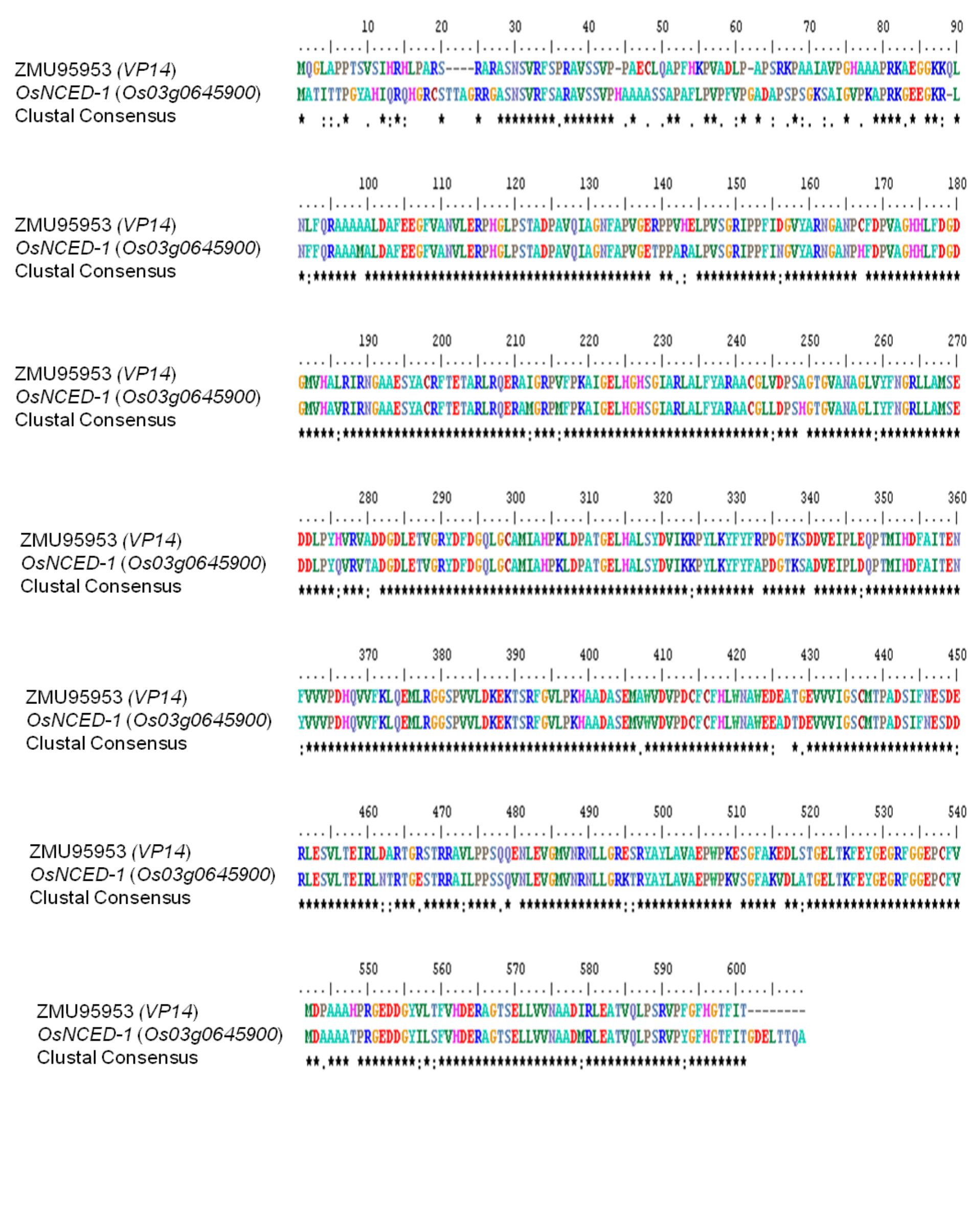

Supplement: Figure S2 — Sequence alignment of the deduced amino acid sequences of the maize VP14 and the rice OsNCED-1. (2.52 MB TIF) [file pgen.1001098.s002.tif]

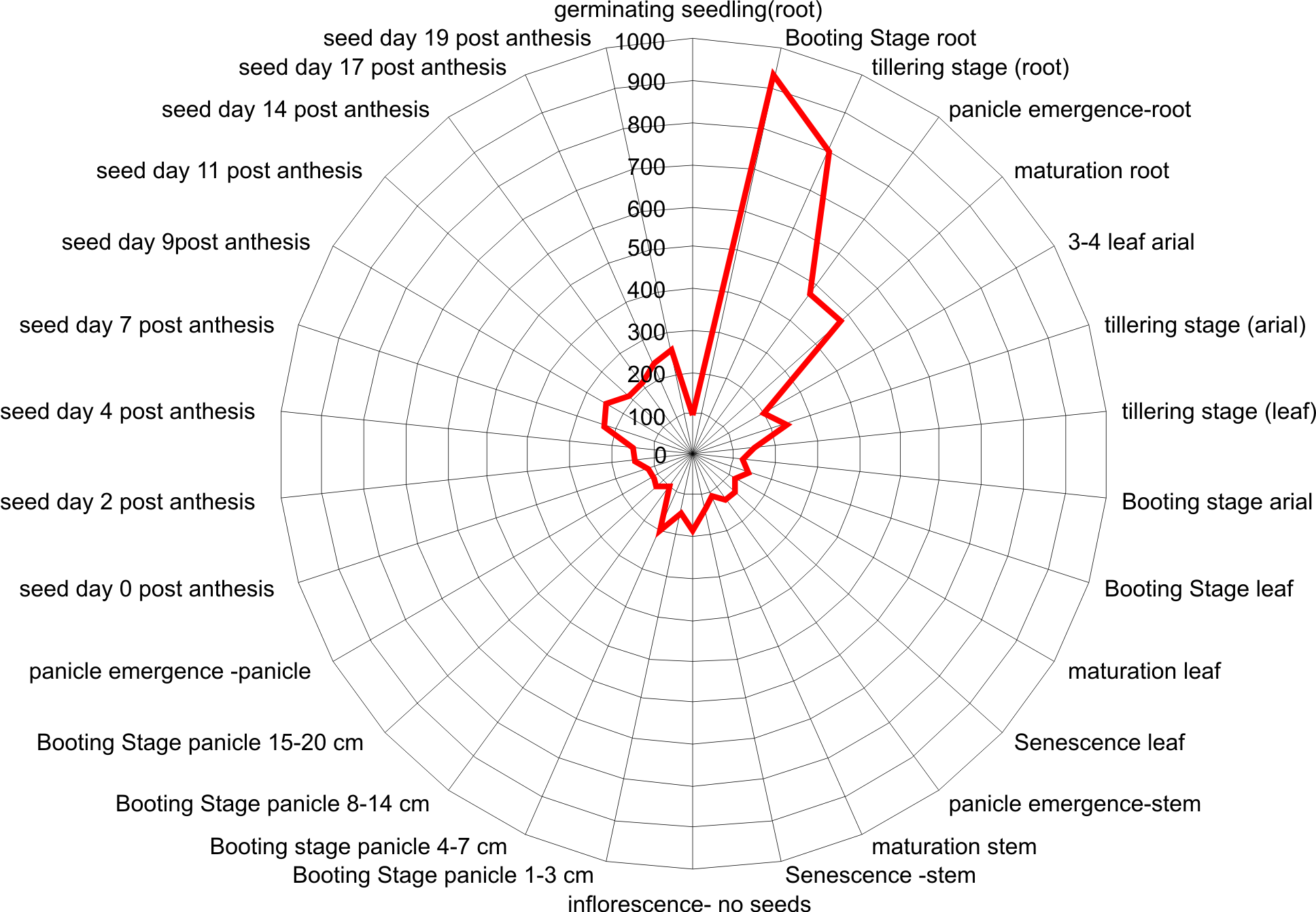

Supplement: Figure S3 — The OsAP2-39-1 expression level measured using microarray technique in various rice plant tissues throughout the plant growth and development. Data present the absolute value of gene expression. (0.61 MB TIF) [file pgen.1001098.s003.tif]

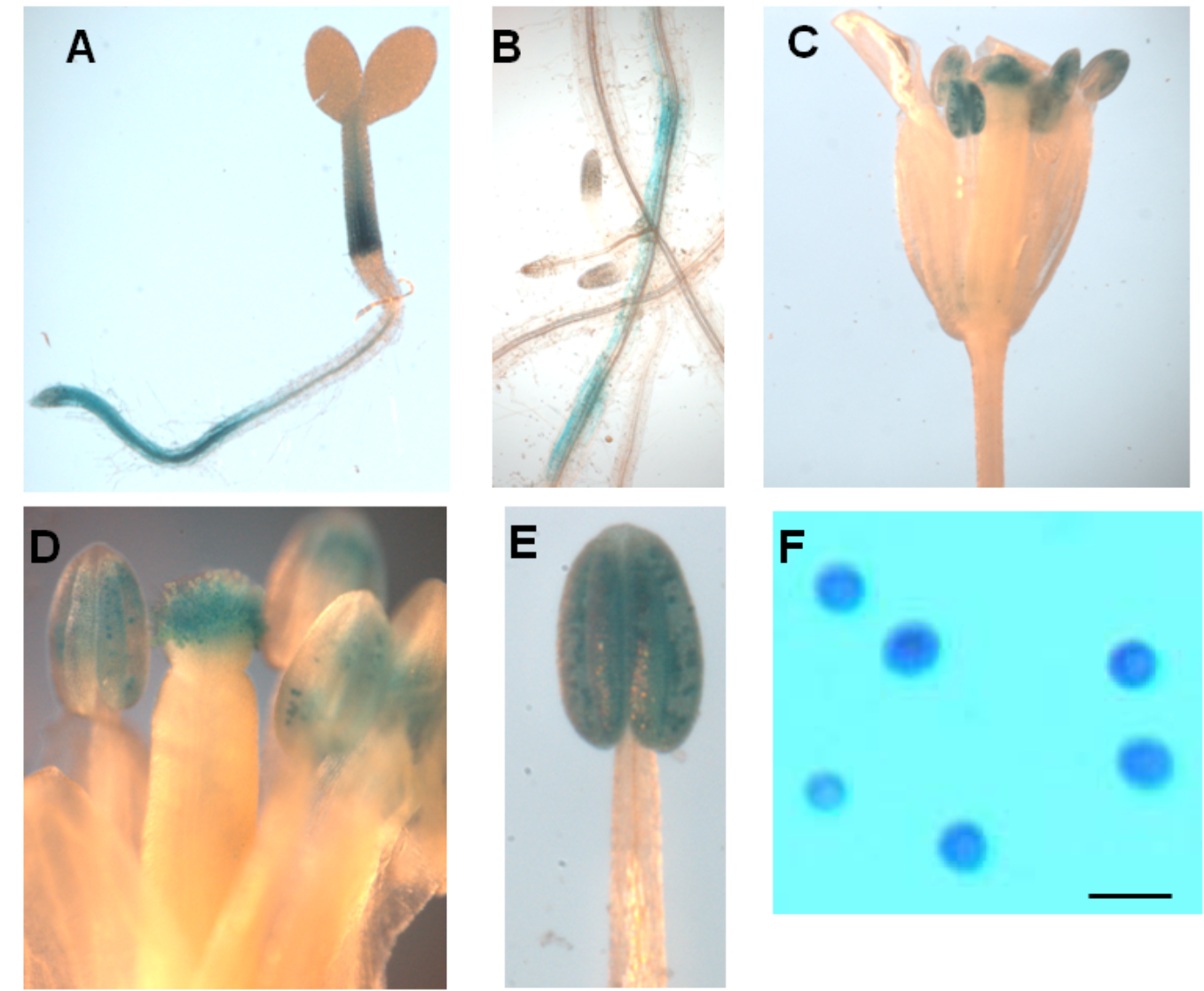

Supplement: Figure S4 — OsNUE39 is expressed in the pollen grains and root of Arabidopsis. The expression pattern of OsNUE39 determined by GUS staining of OsNUE39 promoter fused to GUS in Arabidopsis Wt. OsNUE39 is localized in the root and hypocotyls of the seedlings (A) and roots of the mature plants (B) and also in the flowers (C). (D) A closer look showing the GUS stain in the anthers and on the stigma during pollination. (E) GUS stains in the anther sac. (F) Dissected pollen stained with GUS. Bar = 2 mm. (1.41 MB TIF) [file pgen.1001098.s004.tif]

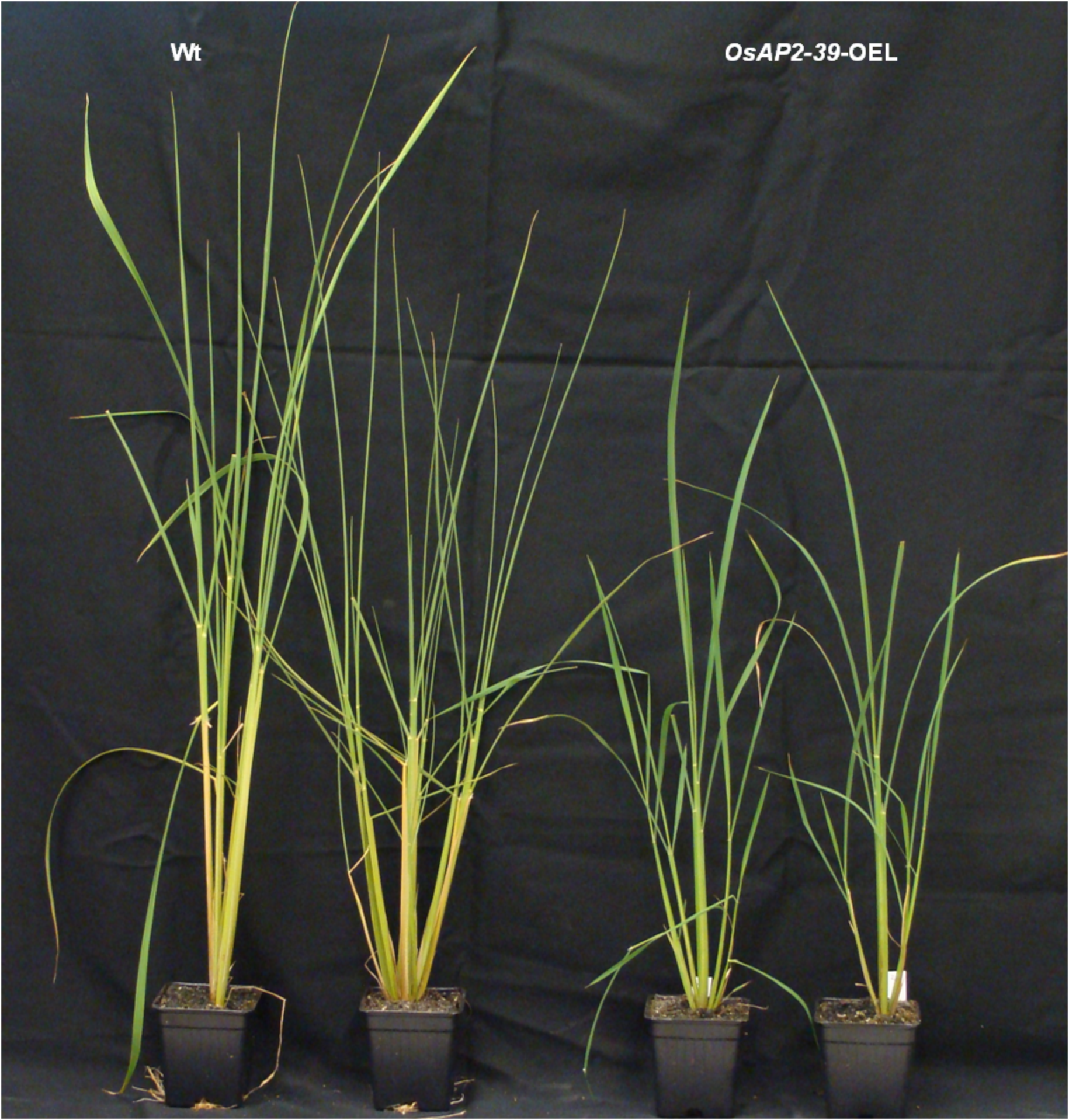

Supplement: Figure S5 — OsAP2-39-1 overexpression lines (OsAP2-39-OEL) dried slower than the wild-type rice pants due to low water consumption. (5.04 MB TIF) [file pgen.1001098.s005.tif]
