# Supplementary material for: The APETALA-2-Like Transcription Factor OsAP2-39 Controls Key Interactions between Abscisic Acid and Gibberellin in Rice
Source: PLoS Genet. 2010 Sep 9;6(9):e1001098. doi: 10.1371/journal.pgen.1001098 (PMC2936520; doi:10.1371/journal.pgen.1001098)
Supplement: Table S1 — Genes expressed differentially in the OsAP2-39 transgenic rice leaves. (0.74 MB DOC) [file pgen.1001098.s006.doc]

**Supplemental Table 1.** Genes Expressed Differentially in the *OsAP2-39* transgenic rice leaves.

| **Probe ID** | **P-value** | **Fold Change (*OsNUE39*/WT)** | **Common** | **Genbank** | **Target description** |
| --- | --- | --- | --- | --- | --- |
| OsAffx.4677.1.S1_s_at | 0.0356 | 0.0518 | Os05g0582000 | LOC_Os05g50500.1 | Expressed protein |
| Os.10583.1.S1_s_at | 0.0371 | 0.0564 | Os06g0141200 | AF171223 | Putative zinc finger protein |
| Os.12606.1.S1_at | 0.0356 | 0.0963 | --- | LOC_Os06g28630.1 | Expressed protein |
| Os.3808.4.S1_x_at | 0.0485 | 0.111 | Os01g0186900 | AK105323 | Transposon protein, putative |
| OsAffx.5807.1.S1_at | 0.047 | 0.133 | --- | LOC_Os08g13840.1 | Putative DNA-binding protein ntwrky3 |
| Os.28394.2.S1_s_at | 0.0371 | 0.14 | Os06g0625400 | AK059029 | Metalloendopeptidase; similar to peptidase M16 family |
| OsAffx.9749.1.S1_at | 0.0344 | 0.151 | Os01g0957800 | NM_001051983 | Putative cytochrome P450 |
| Os.28983.1.S1_at | 0.0361 | 0.156 | Os07g0162700 | AK110810 | Encodes a gibberellin (GA) receptor ortholog of the rice GA receptor gene (osgid1). Has GA-binding activity, showing higher affinity to GA4. Interacts with DELLA proteins in vivo in the presence of GA4. |
| Os.57569.2.A1_s_at | 0.0467 | 0.156 | Os08g0139700 | CB617889 | Putative translation initiation factor |
| Os.15841.1.S1_a_at | 0.0386 | 0.159 | Os11g0150400 | AK058243 | Encodes a heat stable protein with antimicrobial and antifungal activity. |
| Os.34249.2.S1_x_at | 0.0361 | 0.161 | --- | NM_191264 | Putative auxin response transcription factor |
| Os.9885.1.S1_x_at | 0.0359 | 0.163 | Os03g0826200 | AK121165 | Similar to thylakoid soluble phosphoprotein [Spinacia oleracea] (GB:CAD45559.1) |
| Os.56075.1.S1_at | 0.0361 | 0.166 | Os03g0126900 | AK109217 | Expressed protein |
| Os.32072.1.S1_at | 0.0457 | 0.17 | Os08g0432500 | AK106170 | Similar to ATP-dependent Clp protease adaptor protein clps [Trichodesmium erythraeum IMS101] ( |
| Os.5495.1.S1_at | 0.0344 | 0.174 | Os04g0450100 | AK100647 | Similar to rhodanese-like domain-containing protein [Arabidopsis thaliana] |
| Os.3454.1.S1_at | 0.0488 | 0.175 | Os01g0919400 | AK121341 | Encodes a protein with putative sucrose-phosphate synthase activity. |
| Os.11846.1.S1_at | 0.0361 | 0.179 | Os05g0424000 | AK065891 | Aap2, amino acid permease 2, t2k12.6 |
| Os.5210.1.S1_at | 0.0417 | 0.182 | Os08g0503700 | AK072183 | Encodes a tonoplast malate/fumarate transporter. |
| Os.26807.1.S1_at | 0.0361 | 0.185 | Os01g0221600 | CB618460 | ALMT1, putative, expressed |
| Os.52346.1.S1_at | 0.0378 | 0.186 | Os05g0526800 | AK066551 | UDP-glucoronosyl/UDP-glucosyl transferase family protein |
| Os.7784.1.S1_at | 0.0417 | 0.187 | Os12g0510900 | AK058220 | WLIM1; transcription factor; similar to LIM domain-containing protein [Arabidopsis thaliana] ( |
| Os.14882.1.S1_at | 0.0378 | 0.188 | Os12g0116600 | AK102093 | Member of WRKY Transcription Factor; Group III |
| OsAffx.3920.1.S1_s_at | 0.0457 | 0.194 | Os04g0344100 | AK241330 | Terpene synthase family, metal binding domain, putative |
| Os.11565.1.S1_at | 0.0344 | 0.194 | Os10g0109300 | AK104730 | Peroxidase 32 (PER32) (P32) (PRXR3); |
| Os.54440.1.S1_at | 0.0428 | 0.197 | Os04g0623200 | AK105231 | Expressed protein |
| Os.12161.1.S1_at | 0.0401 | 0.2 | Os01g0703400 | D85317 | Encodes a protein with farnesyl diphosphate synthase activity. |
| OsAffx.15220.1.S1_s_at | 0.0378 | 0.2 | --- | LOC_Os01g19310.1 | Retrotransposon protein, putative, LINE subclass |
| Os.34960.1.S1_at | 0.0371 | 0.205 | Os07g0691300 | AK069499 | Similar to wound-responsive protein-related [Arabidopsis thaliana] |
| Os.7602.1.S1_at | 0.0378 | 0.206 | Os02g0658800 | AK059638 | Member of BETA-EXPANSINS. Naming convention from the Expansin Working Group |
| Os.7144.2.S1_a_at | 0.0378 | 0.214 | Os05g0405000 | AK103898 | Ppdk (pyruvate orthophosphate dikinase) |
| Os.11143.1.S1_at | 0.0371 | 0.221 | Os03g0729600 | AK063169 | Expressed protein |
| Os.16902.1.S1_at | 0.047 | 0.225 | Os02g0628200 | AK070820 | Integral membrane protein like, putative, expressed |
| Os.9708.1.S1_at | 0.0387 | 0.226 | Os06g0549300 | AU086033 | Reticuline oxidase precursor, putative, expressed |
| Os.48986.1.S1_s_at | 0.0344 | 0.227 | --- | LOC_Os05g04000.1 | Expressed protein |
| Os.11929.1.S1_at | 0.0344 | 0.232 | Os03g0583800 | AK064786 | Peroxisomal membrane protein-related |
| Os.9685.1.S1_a_at | 0.0386 | 0.236 | Os09g0419200 | AK067949 | Similar to Eucalyptus gunnii alcohol dehydrogenase of unknown physiological function |
| Os.9885.2.S1_x_at | 0.0386 | 0.237 | Os03g0826200 | BI799434 | Similar to thylakoid soluble phosphoprotein [Spinacia oleracea] |
| Os.47702.1.S1_at | 0.0401 | 0.238 | Os08g0351200 | NM_001068147 | Expressed protein |
| Os.5179.1.A1_at | 0.0356 | 0.239 | Os08g0465700 | AK072861 | Cytochrome p450 |
| Os.11146.1.S1_at | 0.0426 | 0.24 | Os05g0541200 | AK068633 | Lysine decarboxylase-like protein, putative, expressed |
| Os.1503.1.S1_at | 0.0361 | 0.244 | Os06g0662200 | AB053474 | Arabidopsis thaliana basic leucine zipper 63, at5g28770.1, atbzip63, bzo2h3, t32b20.4, t32b20_4 |
| Os.6102.1.S1_s_at | 0.0371 | 0.247 | Os06g0607100 | AU166391 | Phosphatidic acid phosphatase-related / PAP2-related; similar to phosphatidic acid phosphatase-related |
| Os.46658.1.S1_at | 0.0457 | 0.248 | Os10g0489500 | NM_001071453 | Putative terpene synthase |
| Os.23224.1.S1_at | 0.0361 | 0.251 | Os06g0517700 | CR288926 | Thionin, putative; Identical to Probable thionin-2.3 precursor |
| Os.50175.2.S1_at | 0.0386 | 0.256 |  | AJ491816 | Putative sodium transporter (hkt4 gene) |
| Os.49319.1.S1_at | 0.0361 | 0.258 | Os02g0769100 | AK107987 | Auxin-responsive protein, putative / small auxin up RNA (SAUR_B) |
| Os.50130.1.S1_at | 0.0401 | 0.259 | Os04g0116800 | AK119922 | Encodes KCS4, a member of the 3-ketoacyl-coa synthase family involved in the biosynthesis of VLCFA (very long chain fatty acids). |
| Os.56279.1.S1_at | 0.0485 | 0.269 | Os08g0118900 | AK109749 | Adenylate kinase family protein; similar to ADK/ATPADK1 (ADENOSINE KINASE), adenylate kinase/ nucleotide kinase [Arabidopsis thaliana] |
| OsAffx.15014.1.S1_s_at | 0.0437 | 0.269 | --- | LOC_Os05g38680.1 | Expressed protein |
| Os.1411.1.S1_at | 0.0388 | 0.279 | Os01g0206700 | AK065589 | Encodes CBL-interacting protein kinase 12 (CIPK12). |
| Os.16221.1.S1_at | 0.0485 | 0.28 | Os10g0521400 | AK109013 | Esterase/lipase/thioesterase family protein; similar to hydrolase, alpha/beta fold family protein [Arabidopsis thaliana] |
| Os.39998.1.A1_x_at | 0.0344 | 0.281 | --- | D42674 | Expressed protein |
| Os.42521.1.S1_at | 0.0376 | 0.282 | Os01g0828100 | CF326471 | Similar to Eucalyptus gunnii alcohol dehydrogenase of unknown physiological function |
| Os.27613.1.A1_at | 0.0344 | 0.282 | Os05g0170900 | CB679962 | Glucose-6-phosphate/phosphate translocator, putative |
| Os.6965.1.S1_at | 0.0371 | 0.284 | Os03g0311000 | AK099476 | Encodes a member of the ERF (ethylene response factor) subfamily B-1 of ERF/AP2 transcription factor family (ATERF-7). The protein contains one AP2 domain. Phosphorylated by PKS3 in vitro. Involved in ABA-mediated responses. Acts as a repressor of GCC box–mediated transcription together with atsin3 and HDA19. |
| Os.45931.1.S1_at | 0.0378 | 0.289 | Os04g0403900 | BI805601 | Expressed protein |
| Os.3766.1.S1_at | 0.0457 | 0.293 | Os01g0797600 | AK069262 | Encodes a member of the ERF (ethylene response factor) subfamily B-1 of ERF/AP2 transcription factor family (ATERF-7). The protein contains one AP2 domain. Phosphorylated by PKS3 in vitro. Involved in ABA-mediated responses. Acts as a repressor of GCC box–mediated transcription together with atsin3 and HDA19. |
| Os.12723.1.S1_at | 0.0378 | 0.294 | Os03g0285700 | AY254495 | Encodes a cytosolic ascorbate peroxidase APX1. Ascorbate peroxidases are enzymes that scavenge hydrogen peroxide in plant cells. |
| Os.10679.1.S1_at | 0.0347 | 0.298 | Os02g0130100 | AK121866 | Encodes a protein predicted to function in tandem with PDX1 to form glutamine amidotransferase complex with involved in vitamin B6 biosynthesis. |
| Os.46725.1.S1_at | 0.039 | 0.298 | Os10g0140700 | CB620435 | Expressed protein |
| Os.10164.1.S1_at | 0.0482 | 0.3 | Os09g0527900 | AK122172 | Salt tolerance-like protein, putative, expressed |
| Os.22651.1.S1_at | 0.0436 | 0.302 | Os04g0657300 | CB624212 | Encodes a protein with farnesyl diphosphate synthase activity. |
| Os.20203.1.S1_s_at | 0.0347 | 0.303 | Os01g0198900 | AK069974 | Expressed protein |
| Os.49917.1.S1_at | 0.0386 | 0.308 | Os04g0349700 | AK119351 | Gene Product Name: verticillium wilt disease resistance protein, putative, expressed |
| Os.11558.1.S1_at | 0.0361 | 0.309 | Os01g0270300 | AK101508 | Peroxidase, putative; Identical to Peroxidase 24 precursor (PER24) [Arabidopsis Thaliana] |
| Os.4999.1.S1_at | 0.0361 | 0.313 | Os06g0716100 | AK071515 | Expressed protein |
| Os.52185.1.A1_at | 0.0361 | 0.315 | Os04g0438600 | AK065524 | GLTP1 (GLYCOLIPID TRANSFER PROTEIN 1); glycolipid binding / glycolipid transporter; similar to GLTP3 (GLYCOLIPID TRANSFER PROTEIN 3) |
| Os.7985.1.S1_at | 0.0485 | 0.316 | Os03g0290300 | AK058242 | Chloroplastic enzyme responsible for the synthesis of 16:3 and 18:3 fatty acids from galactolipids, sulpholipids and phosphatidylglycerol. |
| Os.17147.1.S1_x_at | 0.0361 | 0.317 | Os06g0228200 | AK112022 | Aquaporin NIP4.1, putative, expressed |
| Os.47907.1.S1_at | 0.046 | 0.319 | Os04g0320700 | AK105783 | Cytokinin-O-glucosyltransferase 2, putative, expressed |
| Os.27539.1.A1_at | 0.041 | 0.32 | Os05g0573200 | AK070042 | Isocitrate dehydrogenase, putative / NADP+ isocitrate dehydrogenase, putative; similar to ICDH, isocitrate dehydrogenase (NADP+) [Arabidopsis thaliana] |
| Os.3163.1.A1_at | 0.04 | 0.322 | Os03g0707200 | CB624422 | Integral membrane protein DUF6 containing protein, expressed |
| Os.52259.1.S1_at | 0.0361 | 0.322 | Os12g0106500 | AK065945 | Nodulin-related; similar to unknown protein [Arabidopsis thaliana] (TAIR:AT5G45275.1); |
| Os.12484.1.S1_at | 0.0356 | 0.323 | Os02g0680700 | CR281424 | Myb family transcription factor; similar to myb family transcription factor [Arabidopsis thaliana] ( |
| Os.12029.1.S1_a_at | 0.0361 | 0.325 | Os06g0157000 | AK066113 | GDSL-motif lipase, putative; similar to GDSL-motif lipase, putative [Arabidopsis thaliana] |
| Os.25236.1.S1_x_at | 0.0345 | 0.327 | Os01g0830100 | AK069755 | NDB2 (NAD(P)H DEHYDROGENASE B2); disulfide oxidoreductase; similar to NDB3, NADH dehydrogenase [Arabidopsis thaliana] |
| Os.3754.1.S1_at | 0.0344 | 0.328 | Os01g0223200 | AK067352 | Alpha-L-fucosidase 2 precursor, putative, expressedgdsl-motif lipase |
| Os.37849.1.A1_at | 0.0386 | 0.329 | Os05g0119300 | AK068289 | Glycine-rich protein A3, putative, RAN-binding, expressed |
| Os.10025.1.S1_a_at | 0.0435 | 0.329 | Os12g0626400 | AK063967 | Phytoene synthase, chloroplast precursor, putative, expressed |
| Os.53319.1.S1_at | 0.0378 | 0.33 | Os12g0632900 | AK071957 | Encodes leucine rich repeat (LRR) kinase. |
| Os.49406.1.S1_at | 0.0466 | 0.335 | Os02g0498300 | AK070515 | Similar to permease-related [Arabidopsis thaliana] |
| Os.12092.1.S1_at | 0.0482 | 0.337 | Os03g0150600 | AK070341 | Encodes an inorganic phosphate transporter. Mutants display enhanced arsenic accumulation. |
| Os.31636.1.S1_at | 0.0417 | 0.337 | Os08g0425500 | AK106998 | I inositol-1,4,5-trisphosphate 5-phosphatase CVP2, putative, expressed |
| Os.53428.1.S1_at | 0.0436 | 0.339 | Os09g0433800 | AK104973 | Senescence-associated protein-related |
| Os.9679.1.S1_at | 0.04 | 0.339 | Os11g0180000 | AK060077 | Dirigent-like protein pdir9, putative, expressed |
| Os.32827.1.S1_at | 0.0463 | 0.343 | Os01g0651800 | AK102737 | Triacylglycerol lipase, putative, expressed |
| Os.37728.1.S1_x_at | 0.0361 | 0.345 | Os08g0489300 | AK104597 | Methyladenine glycosylase family protein; |
| Os.8314.1.S1_at | 0.0467 | 0.347 | Os04g0689500 | AK060983 | Expressed protein |
| Os.39998.1.A1_at | 0.0371 | 0.348 | --- | D42674 | Expressed protein |
| Os.12943.1.S1_at | 0.0361 | 0.349 | --- | LOC_Os06g47294.1 | Dehydration-responsive protein-like |
| Os.11789.1.S1_at | 0.04 | 0.35 | Os03g0109300 | AK099538 | Carboxy-lyase, putative, expressed |
| Os.56275.1.S1_x_at | 0.0386 | 0.35 | Os10g0560400 | AK109732 | Zinc finger (B-box type) family protein; Identical to Zinc finger protein CONSTANS-LIKE 4 (COL4) [Arabidopsis Thaliana] |
| Os.6960.1.S1_at | 0.0361 | 0.351 | Os06g0600400 | AK106672 | Encodes a member of the cytochrome p450 family that serves as a control point between multiple photoreceptor systems and brassinosteroid signal transduction. |
| Os.10901.1.S1_a_at | 0.0371 | 0.356 | Os08g0157600 | AY452478 | Myb-related putative transcription factor involved in circadian rhythm along with another myb transcription factor CCA1, LATE ELONGATED HYPOCOTYL, LATE ELONGATED HYPOCOTYL 1, LHY, LHY1, T25K16.6, T25K16_6 |
| Os.33762.1.S1_at | 0.0378 | 0.358 | Os01g0209800 | AK099094 | Encodes a member of the cationic amino acid transporter (CAT) subfamily of amino acid polyamine choline transporters |
| Os.10322.1.S1_s_at | 0.0361 | 0.36 | Os07g0569100 | NM_001066571 | Expressed protein |
| Os.6998.1.S1_at | 0.0371 | 0.363 | Os03g0571900 | CF304368 | Transparent testa 12 protein, putative, expressed |
| Os.9220.2.S1_x_at | 0.0455 | 0.364 | Os10g0378300 | AK069342 | Encodes a copper binding protein that forms tetramers in vitro. Gene is expressed in all tissues examined and protein is localized to the chloroplast. |
| Os.10754.1.S1_at | 0.0437 | 0.365 | Os03g0161200 | AK066932 | Sulfate transmembrane transporter activity |
| Os.9494.1.S1_s_at | 0.047 | 0.365 | Os04g0532400 | CF292249 | Short-chain dehydrogenase/reductase (SDR) family protein; similar to short-chain dehydrogenase/reductase (SDR) family protein [Arabidopsis thaliana] (TAIR:AT5G51030.1); |
| Os.53103.1.S1_at | 0.0449 | 0.366 | Os05g0397300 | AK070832 | Expressed protein |
| Os.7930.1.S1_x_at | 0.0347 | 0.366 | Os08g0478700 | AK058489 | Mitochondrial substrate carrier family protein; similar to mitochondrial substrate carrier family protein [Arabidopsis thaliana] |
| Os.16325.1.S1_at | 0.0347 | 0.368 | Os03g0574900 | AK068853 | Potassium transporter |
| Os.11338.1.S1_at | 0.0457 | 0.37 | Os01g0919600 | AK066408 | Lysine ketoglutarate reductase trans-splicing related 1, putative, expressed |
| Os.14395.1.S1_at | 0.0361 | 0.371 | Os01g0171800 | AK067987 | Expressed protein |
| Os.12035.1.S1_at | 0.0371 | 0.371 | Os02g0734300 | AK059780 | Similar to ATNUDT21 (Arabidopsis thaliana Nudix hydrolase homolog 21), hydrolase [Arabidopsis thaliana] (TAIR:AT1G73540.1); |
| OsAffx.23371.1.S1_x_at | 0.04 | 0.373 | Os01g0346400 | NM_001049497 | Hydroxyproline-rich glycoprotein-like, transposon protein, putative, |
| Os.22781.1.S1_at | 0.0344 | 0.373 | Os02g0601300 | AK099086 | GAPCP-1; glyceraldehyde-3-phosphate dehydrogenase; similar to GAPCP-2, glyceraldehyde-3-phosphate dehydrogenase [Arabidopsis thaliana] |
| Os.11617.1.S1_at | 0.0429 | 0.375 | Os05g0566400 | AK100389 | Member of MAP Kinase |
| Os.17941.2.S1_x_at | 0.0462 | 0.376 | Os03g0127900 | NM_001055370 | Putative potassiumproton antiporter-likeprotein |
| Os.3206.1.S1_at | 0.0494 | 0.378 | Os10g0467800 | AK072259 | Encodes a cellulose synthase isomer. |
| Os.1215.1.S1_at | 0.0361 | 0.38 | Os04g0447800 | AB056061 | Glutamate decarboxylase, putative, expressed |
| Os.11388.1.S1_at | 0.0426 | 0.38 | Os11g0283500 | AK105635 | Proton-dependent oligopeptide transport (POT) family protein |
| OsAffx.16106.1.S1_at | 0.0435 | 0.381 | Os07g0152000 | NM_001065440 | Putative transcription factor PCF6 |
| Os.5160.1.S1_at | 0.0467 | 0.382 | Os07g0656700 | AU173917 | Similar to structural constituent of ribosome [Arabidopsis thaliana] ( |
| Os.13543.1.S1_at | 0.0378 | 0.384 | Os12g0421000 | AK071491 | Heavy-metal-associated domain-containing protein; similar to heavy-metal-associated domain-containing protein [Arabidopsis thaliana] |
| Os.10352.1.S1_at | 0.0378 | 0.385 | Os05g0105000 | AK121285 | Arabidopsis thaliana methionine S-methyltransferase, an enzyme that catalyzes S -methylmethionine formation. |
| Os.11045.1.S1_s_at | 0.0359 | 0.385 | Os05g0169000 | AF435817 | Encodes a thioredoxin-like disulfide reductase. |
| Os.10672.1.S1_at | 0.0347 | 0.387 | Os03g0197000 | AK071163 | Integral membrane protein |
| Os.23471.1.S1_at | 0.0371 | 0.387 | Os11g0286800 | AK072702 | Encodes a multifunctional 2-3-oxidosqualene (OS)-triterpene cyclase that can cyclize OS into lupeol, alpha- and beta-amyrin. |
| OsAffx.28714.1.S1_x_at | 0.0436 | 0.389 | Os07g0509800 | NM_001066285 | Adenosine 5'-phosphosulfate reductase 2 [Zea mays] |
| Os.27591.1.A1_at | 0.0344 | 0.391 | Os04g0201600 | CB679151 | Amino acid transporter family protein; similar to amino acid transporter family protein [Arabidopsis thaliana] |
| Os.5585.1.S1_at | 0.0411 | 0.392 | Os04g0638700 | AK062886 | Expressed protein |
| Os.27662.1.S1_at | 0.0359 | 0.393 | Os12g0615800 | AK073946 | D111/G-patch domain-containing protein; similar to D111/G-patch domain-containing protein [Arabidopsis thaliana] |
| Os.49307.1.S1_at | 0.0378 | 0.394 | Os12g0411700 | AK067639 | ABC transporter family protein; Identical to White-brown complex homolog protein 3 (WBC3) [Arabidopsis Thaliana] |
| Os.7832.1.S1_at | 0.0378 | 0.397 | Os03g0121200 | AK103660 | Peroxidase 30 (PER30) (P30) (PRXR9); Identical to Peroxidase 30 precursor (PER30) [Arabidopsis Thaliana] |
| Os.12766.1.S1_at | 0.04 | 0.399 | Os03g0249700 | AK120462 | Integral membrane protein |
| Os.7665.1.S1_at | 0.0282 | 0.399 | Os04g0657500 | AK066638 | Lipase class 3 family protein; similar to unknown protein [Arabidopsis thaliana] ( |
| Os.17959.1.S1_a_at | 0.0359 | 0.401 | Os08g0440800 | AF357884 | Encodes a mitochondrial succinic semialdehyde dehydrogenase (SSADH). Nomenclature according to Kirch, et al (2004). |
| Os.53722.1.S1_at | 0.0435 | 0.403 | Os03g0426800 | AK099511 | MUB5 (MEMBRANE-ANCHORED UBIQUITIN-FOLD PROTEIN 5 PRECURSOR); Identical to Membrane-anchored ubiquitin-fold protein 5 precursor (MUB5) [Arabidopsis Thaliana] |
| Os.2901.1.S1_at | 0.0388 | 0.405 | Os03g0757200 | AK071136 | Similar to ATUGT85A3 (UDP-GLUCOSYL TRANSFERASE 85A3), glucuronosyltransferase/ transcription factor/ transferase, transferring glycosyl groups [Arabidopsis thaliana] |
| Os.23252.1.S1_at | 0.0371 | 0.405 | Os10g0554900 | AK120846 |  |
| OsAffx.27278.1.S1_x_at | 0.0365 | 0.407 | Os05g0497200 | NM_001062476 | Similar to ethylene responsive element binding factor, putative |
| Os.46151.1.S1_at | 0.0386 | 0.408 | Os10g0506100 | AK120420 | Heavy-metal-associated domain-containing protein; similar to heavy-metal-associated domain-containing protein [Arabidopsis thaliana] |
| Os.5314.1.S1_at | 0.0471 | 0.409 | Os02g0300700 | AK069347 | Eukaryotic translation initiation factor 1A, putative / eif-1A, putative / eif-4C, putative; similar to eukaryotic translation initiation factor 1A, putative / eif-1A, putative / eif-4C, putative |
| Os.37752.1.S1_at | 0.0344 | 0.409 | Os02g0666200 | AF022737 | A member of the plasma membrane intrinsic protein subfamily PIP1 |
| Os.48485.1.S1_at | 0.0482 | 0.411 | Os02g0706400 | AK121386 | MEE3 (maternal effect embryo arrest 3); DNA binding / transcription factor; similar to DNA binding / transcription factor [Arabidopsis thaliana] ( |
| Os.27226.1.S1_at | 0.0361 | 0.411 | Os03g0389100 | AK063170 | ATMC2 (METACASPASE 2); caspase; similar to LOL3 (LSD ONE LIKE 3), caspase/ cysteine-type endopeptidase [Arabidopsis thaliana] |
| OsAffx.24981.1.S1_at | 0.0469 | 0.412 | Os03g0149300 | AK242783 | Integral membrane protein |
| Os.21280.1.S1_at | 0.0457 | 0.415 | Os12g0441600 | BQ908479 | O-methyltransferase, putative; similar to O-methyltransferase family 2 protein [Arabidopsis thaliana] |
| Os.9847.1.S1_at | 0.0347 | 0.416 | Os05g0136900 | AK069868 | Transferase family protein; similar to transferase family protein [Arabidopsis thaliana] |
| Os.3850.3.S1_at | 0.0371 | 0.417 | Os01g0214800 | AK069801 | Lipase, putative; similar to lipase, putative [Arabidopsis thaliana] |
| Os.12623.1.S1_at | 0.0417 | 0.417 | Os01g0892600 | AK104609 | Carboxylesterase; similar to pectinacetylesterase, putative [Arabidopsis thaliana] |
| Os.34767.2.S1_s_at | 0.0485 | 0.417 | Os03g0226400 | NM_001055974 | Putative cation diffusion facilitator 8 |
| Os.9515.1.S1_at | 0.0485 | 0.417 | Os07g0185100 | AK060552 | Putative mitogen activated protein kinase kinase , Serine/threonine protein kinase, whose transcription is regulated by circadian rhythm. |
| Os.25531.1.S1_at | 0.0378 | 0.418 | Os04g0359100 | AK069384 | Chloroplastic quinone-oxidoreductase, putative, expressed, ARP protein (REF); similar to oxidoreductase, zinc-binding dehydrogenase family protein [Arabidopsis thaliana] |
| Os.11129.1.S1_at | 0.0344 | 0.419 | Os10g0502400 | AB011416 | Glutamyl-trna reductase, chloroplast precursor, putative, expressed, Encodes a protein with glutamyl-trna reductase (glutr) activity, catalyzing the NADPH-dependent reduction of Glu-trna(Glu) to glutamate 1-semialdehyde (GSA) with the release of free trna(Glu). It is involved in the early steps of chlorophyll biosynthesis. |
| Os.17814.2.S1_x_at | 0.0376 | 0.42 | Os07g0630800 | AK068064 | Malate dehydrogenase, glyoxysomal precursor, putative, expressed, Encodes a protein with NAD-dependent malate dehydrogenase activity, located in chloroplasts. |
| Os.27682.1.S1_at | 0.0426 | 0.421 | Os08g0243500 | AK068915 | Encodes NADPH-cytochrome P450 reductase that catalyzes the first oxidative step of the phenylpropanoid general pathway. |
| Os.16884.1.S1_at | 0.04 | 0.423 | Os03g0717900 | AK099485 | Integral membrane protein |
| Os.18307.1.S1_at | 0.0455 | 0.423 | Os07g0577500 | AK100831 | Trichohyalin-related; similar to auxilin-related [Arabidopsis thaliana] |
| Os.13993.1.S1_at | 0.0393 | 0.423 | Os08g0434300 | AK058477 | Encodes a protein with NAD-dependent malate dehydrogenase activity, located in chloroplasts. |
| Os.11623.1.S1_a_at | 0.0361 | 0.423 | Os10g0341700 | AK069071 | Atcslb02, atcslb2, cellulose synthase like b2, cslb02, t26b15.18, t26b15_18 |
| Os.52498.1.S1_at | 0.0488 | 0.424 | Os01g0555200 | AK067541 | Expressed protein |
| Os.1502.1.S1_at | 0.0386 | 0.425 | Os02g0175100 | AB053473 | Encodes a basic leucine zipper (bzip) transcription factor atbzip10. Atbzip10 shuttles between the nucleus and the cytoplasm. |
| Os.20617.2.S1_x_at | 0.0344 | 0.425 | Os07g0656200 | NM_001067037 | Beta-glucosidase |
| Os.17889.1.S1_at | 0.0344 | 0.428 | Os05g0541000 | AK072284 | Expressed protein |
| Os.23800.2.S1_x_at | 0.0485 | 0.429 | Os02g0216500 | AK103179 | Expressed protein |
| Os.11330.2.S1_x_at | 0.0361 | 0.429 | Os07g0448800 | AB029446 | Member of the plasma membrane intrinsic protein subfamily PIP2. Localizes to the plasma membrane and exhibits water transport activity in Xenopus oocyte |
| Os.10524.2.S1_at | 0.0356 | 0.429 | Os12g0124000 | AK060098 | Forkhead-associated domain-containing protein / FHA domain-containing protein; similar to unnamed protein product |
| Os.49527.1.S1_at | 0.0378 | 0.431 | Os03g0130100 | AK110783 | Encodes a benzoate-coa ligase. Involved in the biosynthesis of benzoyloxyglucosinolate in Arabidopsis seeds. |
| Os.38748.1.S1_s_at | 0.0359 | 0.431 | Os11g0642800 | CA762966 | Encodes a protein with similarity to glutathione synthetases, which catalyzes one of the early steps in glutathione biosynthesis. |
| Os.17681.1.S1_at | 0.0457 | 0.433 | Os04g0454200 | AK072059 | Monosaccharide transporter, putative; Identical to Sugar transport protein 5 (STP5) [Arabidopsis Thaliana] |
| Os.5643.1.S1_at | 0.0454 | 0.434 | Os01g0869200 | AK073453 | Magnesium transporter cora-like family protein (MRS2-3); similar to magnesium transporter cora-like family protein (MRS2-1) [Arabidopsis thaliana] |
| Os.53467.1.S1_at | 0.0426 | 0.434 | Os03g0342100 | AK072786 | Integral membrane protein |
| Os.23417.1.A1_at | 0.0455 | 0.435 | Os06g0314600 | AK067321 | A member of the Arabidopsis SABATH methyltransferase gene family. Encodes NAMT1, a methyltransferase that methylates nicotinic acid. |
| Os.8338.1.S1_at | 0.0379 | 0.435 | Os08g0549300 | CB631913 | Acp5, acyl carrier protein 5, a_tm021b04.6, t21b4.110, t21b4_110 |
| Os.5659.1.S1_at | 0.038 | 0.436 | Os02g0757700 | AK069370 | Ubiquitin-protein ligase, putative, expressed |
| Os.27657.1.A1_at | 0.0371 | 0.436 | Os04g0588600 | CB682256 | Encodes an ATP-dependent MRP-like ABC transporter able to transport glutathione-conjugates as well as chlorophyll catabolites. |
| Os.27451.1.S1_at | 0.0371 | 0.437 | Os06g0680500 | AK099745 | Member of Putative ligand-gated ion channel subunit family |
| Os.6672.1.S1_at | 0.0361 | 0.438 | Os01g0283300 | AK068117 | Tyrosine-protein phosphatase non-receptor type 23, putative, expressed |
| Os.27035.1.S1_s_at | 0.0361 | 0.438 | Os01g0563000 | AK067665 | Peptidyl-prolyl cis-trans isomerase, putative / FK506-binding protein, putative; similar to ROF1 (ROTAMASE FKBP 1), FK506 binding / calmodulin binding / peptidyl-prolyl cis-trans isomerase [Arabidopsis thaliana] |
| Os.8259.1.S1_at | 0.0401 | 0.439 | Os05g0382900 | AK061728 | Annexin-like protein RJ4, putative, expressed |
| Os.53190.1.S1_at | 0.0387 | 0.439 | Os07g0613500 | AK071281 | Protein kinase, putative; similar to protein kinase, putative [Arabidopsis thaliana] |
| OsAffx.19658.1.S1_at | 0.0361 | 0.439 | Os12g0240900 | NM_001073009 | Similar to caffeic acid O-methyltransferase |
| Os.18305.1.S2_at | 0.0457 | 0.441 | Os05g0582300 | AK066579 | Encodes a protein with solanesyl diphosphate synthase activity. |
| Os.26767.1.A1_at | 0.0416 | 0.441 | Os11g0470500 | CB633345 | Leucine-rich repeat family protein / protein kinase family protein; similar to leucine-rich repeat family protein / protein kinase family protein [Arabidopsis thaliana] |
| Os.11854.1.S1_at | 0.0376 | 0.442 | Os03g0704100 | AK070474 | Plastid-lipid associated protein PAP, putative; Identical to Probable plastid-lipid-associated protein 3, chloroplast precursor (PAP3) [Arabidopsis Thaliana] |
| Os.9851.1.S1_at | 0.0485 | 0.443 | Os01g0672100 | AK102794 | ANAC010 (Arabidopsis NAC domain containing protein 10) |
| OsAffx.12451.1.S1_x_at | 0.0455 | 0.444 | Os02g0633300 | NM_001054044 | Oligopeptide transporter OPT-like |
| Os.52602.1.S1_x_at | 0.0378 | 0.445 | Os10g0510500 | AK068270 | Auxin-responsive family protein; similar to auxin-responsive family protein [Arabidopsis thaliana] |
| Os.20354.1.S1_at | 0.0465 | 0.446 | Os03g0656500 | AK121052 | Cation exchanger, putative; similar to CAX9 (CATION EXCHANGER 9), cation:cation antiporter [Arabidopsis thaliana] |
| Os.27860.1.A1_at | 0.0361 | 0.447 | Os01g0739200 | AK073172 | Dual specificity protein phosphatase family protein; similar to dual specificity protein phosphatase family protein [Arabidopsis thaliana] |
| Os.27615.1.S1_at | 0.0426 | 0.451 | Os03g0216700 | AK101556 | MATE efflux protein-related; similar to MATE efflux family protein [Arabidopsis thaliana] |
| Os.12225.1.S1_at | 0.0436 | 0.451 | Os04g0500700 | AK119237 | Anthranilate N-benzoyltransferase protein 2, putative, expressed |
| Os.50635.1.S1_x_at | 0.0365 | 0.451 | --- | NM_001050321 | Putative aspartic protease |
| Os.8012.1.S1_at | 0.0455 | 0.452 | Os08g0101700 | AK061172 | Similar to ACYB-2 (Arabidopsis cytochrome b561 -2), carbon-monoxide oxygenase [Arabidopsis thaliana] |
| Os.23712.2.S1_at | 0.0455 | 0.454 | Os08g0280100 | AK072150 | ATPAP13/PAP13; acid phosphatase; similar to ATPAP15/PAP15 (purple acid phosphatase 15), acid phosphatase/ protein serine/threonine phosphatase [Arabidopsis thaliana] |
| Os.26842.1.S1_at | 0.0361 | 0.456 | Os01g0764400 | AK068983 | Arabidopsis thaliana chorismate mutase 1, atcm1, chorismate mutase 1, cm1, mxo21.4 |
| Os.17250.1.S1_x_at | 0.0361 | 0.456 | Os10g0469900 | AK105737 | Peptide transporter PTR2, putative, expressed |
| Os.27603.1.S1_at | 0.0344 | 0.459 | Os01g0356000 | NM_001049535 | Putative multi resistance protein ABC transporter |
| Os.15593.1.S1_at | 0.0356 | 0.46 | Os03g0263900 | AK121215 | Calcium ion binding protein, putative, expressed, Encodes a N-acetyl glucosamine transferase that may glycosylate other molecules involved in GA signaling. Contains a tetratricopeptide repeat region, and a novel carboxy-terminal region. SPY acts as both a repressor of GA responses and as a positive regulation of cytokinin signalling. SPY may be involved in reducing ROS accumulation in response to stress. |
| Os.27805.1.S1_at | 0.0361 | 0.46 | Os03g0684400 | AK100086 | Atmgt10, gmn10, magnesium (mg) transporter 10, mrn17.6, mrn17_6 |
| OsAffx.23005.1.S1_at | 0.0386 | 0.461 | Os06g0133200 | AK108349 | Expressed protein |
| Os.34249.1.S1_at | 0.0417 | 0.462 | Os01g0753500 | AK072330 | Encodes an auxin response factor. |
| Os.2293.1.S1_at | 0.0371 | 0.462 | Os03g0650000 | AK070258 | YABBY gene family member, likely has transcription factor activity, involved in specifying abaxial cell fate. |
| Os.11394.1.S1_at | 0.0455 | 0.462 | Os12g0105300 | AK072914 | Actin binding protein required for normal chloroplast positioning |
| Os.2528.1.S1_x_at | 0.0359 | 0.462 | --- | LOC_Os10g37500.1 | Atpase, AAA family protein, expressed [ |
| Os.16788.1.S1_at | 0.0361 | 0.463 | Os03g0837300 | AK121140 | Nicotinate phosphoribosyltransferase; similar to nicotinate phosphoribosyltransferase family protein / naprtase family protein [Arabidopsis thaliana] |
| Os.37616.1.S1_at | 0.0386 | 0.463 | Os08g0500700 | AY077617 | Heat shock protein 81-1, putative, expressed, Encodes a chloroplast-targeted 90-kda heat shock protein located in the stroma involved in red-light mediated deetiolation response. |
| Os.14381.2.S1_x_at | 0.0494 | 0.464 | Os03g0401100 | CA754177 | Protein kinase family protein; similar to protein kinase family protein [Arabidopsis thaliana] |
| Os.9787.1.S1_at | 0.0492 | 0.464 | Os07g0500300 | AK120268 | C2 domain-containing protein; similar to C2 domain-containing protein [Arabidopsis thaliana] |
| Os.5209.2.S1_at | 0.0371 | 0.464 | Os09g0518900 | AK072714 | Expressed protein |
| Os.49658.1.S1_at | 0.0344 | 0.465 | Os05g0534400 | AK101368 | Encodes a member of the Arabidopsis CBL (Calcineurin B-like Calcium Sensor) protein family. |
| Os.5387.1.S1_x_at | 0.0465 | 0.465 | Os10g0521500 | AK105796 | Hydrolase, alpha/beta fold family protein; similar to hydrolase, alpha/beta fold family protein [Arabidopsis thaliana] |
| Os.11160.1.S2_at | 0.0344 | 0.468 | Os05g0208000 | AK071500 | Mitochondrial substrate carrier family protein; similar to mitochondrial substrate carrier family protein [Arabidopsis thaliana] (TAIR:AT4G24570.1); |
| Os.2488.1.S1_at | 0.0437 | 0.469 | Os03g0230500 | AK100971 | ATITPK4 (INOSITOL 1,3,4-TRISPHOSPHATE 5/6-KINASE 4); catalytic; Identical to Inositol-tetrakisphosphate 1-kinase 4 (ITPK4) [Arabidopsis Thaliana] |
| OsAffx.4957.1.S1_s_at | 0.0401 | 0.471 | Os06g0483200 | NM_001064189 | Similar to beta-amyrin synthase |
| Os.57545.1.S1_x_at | 0.0426 | 0.473 | Os02g0729700 | AF264731 | Encodes a class I hdzip (homeodomain-leucine zipper) protein that is a positive regulator of ABA-responsiveness, mediating the inhibitory effect of ABA on growth during seedling establishment. |
| Os.26965.1.S1_at | 0.0356 | 0.474 | Os01g0826900 | AK105709 | Expressed protein |
| Os.13926.1.S1_at | 0.0457 | 0.474 | Os07g0695800 | AK059174 | 2-oxoglutarate dehydrogenase E1 component, mitochondrial precursor, putative, expressed |
| Os.52298.1.S1_at | 0.0455 | 0.475 | Os06g0120200 | AK066196 | Expressed protein |
| Os.52568.1.S1_at | 0.0371 | 0.475 | Os12g0121000 | AK068049 | Expressed protein |
| Os.7594.1.S1_at | 0.0411 | 0.478 | Os05g0499400 | AK068896 | Peroxidase, putative; Identical to Peroxidase 56 precursor (PER56) [Arabidopsis Thaliana] (GB:Q9LXG3;GB:Q41955); similar to peroxidase 27 (PER27) (P27) (PRXR7) [Arabidopsis thaliana] ( |
| Os.50442.1.S1_at | 0.0466 | 0.479 | Os03g0124000 | AK121207 | Encodes a member of the BEL family of homeodomain proteins. Plants doubly mutant for saw1/saw2 (blh2/blh4) have serrated leaves. |
| Os.7972.1.S1_at | 0.0372 | 0.481 | Os02g0611200 | AK070269 | Encodes a S-adenosylmethionine decarboxylase involved in polyamine biosynthesis. |
| Os.8504.1.S1_at | 0.0361 | 0.481 | Os04g0502800 | AK099877 | Nodulin family protein; similar to nodulin family protein [Arabidopsis thaliana] |
| Os.26479.1.A1_a_at | 0.0361 | 0.482 | Os01g0953600 | AK071823 | Encodes NAD(P)H:quinone reductase which is an FMN binding protein that catalyzes the reduction of quinone substrates to hydroquinones. |
| Os.27308.1.S1_at | 0.0432 | 0.482 | Os05g0455200 | AK063788 | Encodes a member of the BEL family of homeodomain proteins. |
| Os.27281.1.S1_at | 0.0361 | 0.483 | Os04g0116600 | AK064965 | Short-chain dehydrogenase/reductase (SDR) family protein |
| Os.17226.1.S1_s_at | 0.0466 | 0.485 | Os01g0645200 | BI812412 | Bile acid:sodium symporter family protein; similar to bile acid:sodium symporter family protein [Arabidopsis thaliana] |
| Os.14248.1.S1_at | 0.0361 | 0.486 | Os03g0180100 | AK108326 | Integral membrane protein |
| Os.10311.1.S1_a_at | 0.0361 | 0.486 | Os06g0254300 | AK066241 | Encodes a calcium binding protein whose mrna is induced upon treatment with nacl, ABA and in response to dessication. |
| Os.9547.1.S1_at | 0.0361 | 0.486 | Os08g0374800 | AB096863 | Encodes a protein with UDP-D-glucose 4-epimerase activity. |
| Os.5186.1.S1_at | 0.0485 | 0.488 | Os04g0406600 | AK103609 | ADT5 (AROGENATE DEHYDRATASE 5); arogenate dehydratase/ prephenate dehydratase; similar to ADT4 (AROGENATE DEHYDRATASE 4), arogenate dehydratase/ prephenate dehydratase [Arabidopsis thaliana] |
| Os.4627.1.S1_x_at | 0.0456 | 0.489 | Os07g0678600 | AK058819 | Encodes a CBL-interacting protein kinase with similarity to SOS protein kinase. |
| Os.56880.1.S1_at | 0.0409 | 0.491 | Os01g0542700 | AK110526 | Common plant regulatory factor 7, putative, expressed |
| Os.17868.1.S1_at | 0.0436 | 0.492 | Os12g0134000 | AK066940 | Hydroxymethylglutaryl-coa lyase, putative / 3-hydroxy-3-methylglutarate-coa lyase, putative / HMG-coa lyase, putative; similar to MAM1 (2-isopropylmalate synthase 3), 2-isopropylmalate synthase [Arabidopsis thaliana] |
| Os.10311.2.A1_x_at | 0.0361 | 0.493 | Os06g0254300 | AK101337 | Encodes a calcium binding protein whose mrna is induced upon treatment with nacl, ABA and in response to dessication. Mrna expression under drought conditions is apparent particularly in leaves and flowers. |
| Os.4244.1.S1_at | 0.0361 | 0.493 | Os08g0498100 | AB110168 | Caffeoyl-coa 3-O-methyltransferase, putative; Identical to Probable caffeoyl-coa O-methyltransferase At4g34050 [Arabidopsis Thaliana] (GB:O49499;GB:Q8L989); similar to caffeoyl-coa 3-O-methyltransferase, putative [Arabidopsis thaliana] |
| Os.20627.3.S1_x_at | 0.0344 | 0.494 | Os07g0520300 | NM_001066336 | Putative 5-alpha-taxadienol-10-beta-hydroxylase |
| OsAffx.17053.1.S1_x_at | 0.0371 | 0.494 | --- | LOC_Os08g17870.1 | Reticulon family protein |
| Os.17941.1.S1_s_at | 0.0361 | 0.495 | Os03g0127900 | AK062977 | Glutathione-regulated potassium-efflux system protein kefb, putative, expressed |
| Os.4161.1.S1_at | 0.04 | 0.496 | Os05g0543400 | AK100273 | Encodes a protein with farnesyl diphosphate synthase activity. |
| Os.12492.1.S1_at | 0.0457 | 0.497 | Os01g0188400 | D16499 | The malic enzyme (EC 1.1.1.40) encoded by atnadp-ME4 is localized to chloroplasts. |
| Os.9901.1.S1_at | 0.0347 | 0.497 | Os03g0351800 | AK070491 | Encodes a putative RING-H2 finger protein RHC1a. |
| Os.26502.1.S1_a_at | 0.0492 | 0.497 | Os04g0434800 | AB114855 | Encodes a chloroplastic stromal ascorbate peroxidase sapx. |
| OsAffx.28032.2.S1_at | 0.0361 | 2.008 | Os06g0612900 | NM_001064602 | Expressed protein |
| Os.11564.1.S1_at | 0.0421 | 2.01 | Os09g0556500 | AK069699 | Trna synthetase class I (C) family protein; similar to trna synthetase class I (C) family protein [Arabidopsis thaliana] |
| OsAffx.3129.1.S1_x_at | 0.0361 | 2.014 | Os03g0137600 | NM_001055438 | Eukaryotic porin |
| Os.10721.1.S1_at | 0.0386 | 2.015 | --- | AK061392 | Expressed protein |
| Os.20176.1.S1_at | 0.0378 | 2.016 | Os03g0747700 | AK058795 | Photosystem II 11 kda protein-related; similar to unnamed protein product [Vitis vinifera] (GB:CAO71168.1) |
| OsAffx.6386.1.S1_at | 0.0344 | 2.023 | --- | AK243615 | Putative ribulose-1,5 bisphosphate carboxylase/oxygenase large subunit N-methyltransferase, chloroplast precursor |
| Os.38077.1.S1_at | 0.0344 | 2.04 | Os01g0589000 | AK099851 | S1 RNA-binding domain-containing protein; similar to RPS1 (ribosomal protein S1), RNA binding |
| Os.39952.1.S1_at | 0.0344 | 2.047 | Os04g0101300 | CB627192 | Chromatin modification-related protein EAF3, putative, expressed, MRG family protein |
| Os.1709.1.S1_x_at | 0.0436 | 2.048 | Os07g0215150 | LOC_Os02g25810.1 | Putative polyprotein |
| Os.5754.1.S1_at | 0.0378 | 2.049 | Os08g0139100 | AK099590 | Plastid developmental protein DAG, putative; similar to plastid developmental protein DAG |
| Os.20200.1.S1_at | 0.0361 | 2.051 | Os07g0490400 | AK067941 | Encodes an immunophilin, FKBP20-2, that belongs to the FK-506 binding protein (FKBP) subfamily functioning as peptidyl-prolyl isomerases (ppiases) in protein folding. |
| Os.27394.1.A1_at | 0.0371 | 2.054 | Os11g0448300 | CB642698 | Expressed protein |
| Os.53046.1.S1_at | 0.0361 | 2.057 | Os01g0940600 | AK070587 | Encodes HCF153, a 15-kda protein involved in the biogenesis of the cytochrome b(6)f complex. Associated with the thylakoid membrane. |
| Os.12608.1.S1_at | 0.0485 | 2.063 | Os07g0158300 | AK067376 | Encodes a nuclear gene with a consensus RNA-binding domain that is localized to the chloroplast. |
| OsAffx.16139.1.S1_at | 0.0386 | 2.067 | Os07g0172600 | NM_001065534 | Selenium-binding protein-like |
| Os.30049.1.S1_at | 0.0361 | 2.074 | Os03g0376600 | AK068720 | Encodes a nuclear gene with a consensus RNA-binding domain that is localized to the chloroplast. |
| Os.7557.2.S1_at | 0.0455 | 2.074 | --- | LOC_Os02g19629.2 | Expressed protein |
| Os.7732.1.S1_at | 0.0435 | 2.077 | Os10g0529800 | CF330701 | Encodes glutathione transferase belonging to the tau class of gsts. |
| Os.14817.1.S1_a_at | 0.0371 | 2.078 | Os10g0512500 | AK067203 | CRS1 / yhby domain containing protein, expressed |
| Os.38791.1.A1_s_at | 0.0361 | 2.082 | Os12g0507500 | CF196059 | SWIB complex BAF60b domain-containing protein |
| Os.46525.2.S1_x_at | 0.0361 | 2.088 | Os10g0198600 | NM_001070831 | Hypothetical protein |
| Os.9540.1.S1_at | 0.0457 | 2.088 | Os10g0571800 | AK121698 | Late embryogenesis abundant protein-related |
| Os.22563.1.S1_at | 0.0361 | 2.105 | Os03g0825500 | AK058472 | Ptac13 (plastid transcriptionally active13) |
| Os.35710.1.S1_at | 0.0435 | 2.108 | Os07g0459000 | AK066176 | Expressed protein |
| Os.23875.1.S1_at | 0.0361 | 2.113 | Os02g0148700 | AK068686 | Expressed protein |
| Os.28459.1.S1_at | 0.0426 | 2.116 | Os11g0448400 | AB095094 | Enodes a subunit of chloroplast RNA polymerase, confers the ability to recognize promoter sequences on the core enzyme. SIG1 is induced by red and blue light. |
| Os.10175.1.S1_at | 0.0426 | 2.12 | Os05g0429900 | D88617 | MYB-type transcription factor (MYB3) that represses phenylpropanoid biosynthesis gene expression |
| Os.49107.1.A1_at | 0.0486 | 2.121 | --- | AK058478 | Expressed protein |
| Os.24860.1.S1_at | 0.0361 | 2.127 | Os03g0616400 | AK071938 | Encodes a chloroplast envelope Ca2+-atpase with an N-terminal autoinhibitor. |
| Os.32449.1.S1_at | 0.0482 | 2.131 | Os02g0744000 | AK064898 | Likely a subunit of the chloroplast NAD(P)H dehydrogenase complex, involved in PSI cyclic electron transport. Located on the thylakoid membrane. Mutant has impaired NAD(P)H dehydrogenase activity. |
| Os.57099.1.S1_at | 0.0361 | 2.146 | Os09g0531100 | AK110919 | Expressed protein |
| Os.51635.1.A1_at | 0.0356 | 2.153 | Os10g0482000 | AK063227 | Expressed protein |
| Os.9792.1.S1_at | 0.0386 | 2.157 | Os02g0152900 | AK061136 | Expressed protein |
| Os.10426.1.S1_at | 0.0347 | 2.168 | Os03g0137600 | AK058690 | Expressed protein |
| Os.11671.1.S1_at | 0.0361 | 2.172 | Os02g0115600 | AK067366 | S1 RNA-binding domain-containing protein; similar to S1 RNA-binding domain-containing protein [Arabidopsis thaliana] (TAIR:AT3G23700.1); |
| Os.20570.1.S1_x_at | 0.0361 | 2.173 | Os06g0115400 | AK112038 | Fe superoxide dismutase whose mrna levels are increased in response to exposure to UV-B. |
| Os.26864.1.S1_at | 0.04 | 2.175 | Os01g0769900 | AK065563 | Plastid transcriptionally active12. Present in transcriptionally active plastid chromosomes. Involved in plastid gene expression. |
| Os.27276.1.S1_at | 0.0436 | 2.183 | --- | CT835325 | Expressed protein |
| Os.52485.1.S1_at | 0.0436 | 2.185 | Os03g0753600 | AK067449 | Expressed protein |
| Os.27394.1.A1_x_at | 0.0361 | 2.204 | Os11g0448300 | CB642698 | Putative protein kinase |
| Os.27069.1.A1_at | 0.0361 | 2.211 | Os03g0160000 | AK068259 | Integral membrane protein |
| Os.18490.3.S1_at | 0.0426 | 2.214 | Os03g0679700 | AK064949 | Encodes a protein involved in thiamin biosynthesis. |
| Os.24663.1.S1_s_at | 0.0493 | 2.224 | Os02g0750400 | AK059134 | Pentatricopeptide (PPR) repeat-containing protein |
| Os.20070.1.S1_x_at | 0.0376 | 2.225 | Os07g0434700 | AK059240 | Encodes a cytoplasmic MAP1 like methionine aminopeptidase which is involved in removing the N-terminal methionine from proteins. |
| Os.23250.1.S1_x_at | 0.0356 | 2.23 | Os10g0555200 | AK068935 | DNA binding / hydrolase, acting on ester bonds / nuclease/ nucleic acid binding / recombinase |
| Os.3774.2.S1_x_at | 0.0435 | 2.232 | Os01g0184500 | NM_001048769 | DEAD-box ATP-dependent RNA helicase 39 dbj|BAD67795.1| putative VASA |
| Os.5639.1.S1_at | 0.0457 | 2.237 | Os08g0540000 | AK070813 | Similar to unknown protein [Arabidopsis thaliana] (TAIR:AT3G59340.1) |
| Os.2685.1.S1_at | 0.04 | 2.239 | Os01g0916600 | AK059446 | Glycine-rich rna-binding protein 3, gr-rbp3, maf19.4, maf19_4 |
| Os.25529.1.S1_at | 0.0387 | 2.247 | Os05g0519600 | AK102422 | 4-methyl-5-thiazole monophosphate biosynthesis protein, putative, expressed |
| Os.46548.2.S1_at | 0.047 | 2.252 | --- | LOC_Os10g39930.2 | Cytochrome P450 family protein |
| OsAffx.3056.1.S1_at | 0.048 | 2.253 | Os02g0774100 | NM_001054807 | Similar to thioredoxin-like 5 |
| OsAffx.4083.1.S1_at | 0.0371 | 2.278 | --- | LOC_Os04g44200.1 | Calcium ion binding [ |
| Os.54940.1.S1_at | 0.0456 | 2.28 | Os04g0522500 | AK107142 | Gene Product Name: gibberellin 2-beta-dioxygenase 7, putative, expressed |
| Os.24034.3.S1_x_at | 0.0467 | 2.281 | Os01g0874800 | AK105063 | 5'-3' exonuclease family protein; similar to 5'-3' exonuclease family protein [Arabidopsis thaliana] |
| Os.52535.1.S1_at | 0.0386 | 2.281 | Os09g0439500 | AK067780 | PSI type II chlorophyll a/b-binding protein (Lhca2*1) mrna, |
| Os.35677.1.S1_at | 0.0479 | 2.286 | Os03g0190100 | AK066332 | Encodes a protein with chlorophyll synthase activity. This enzyme has been shown to perform the esterification of chlorophyllide (a and b), the last step of chlorophyll biosynthesis. |
| OsAffx.7765.1.S1_at | 0.0436 | 2.286 | --- | LOC_Os12g32280.1 | SWIB complex BAF60b domain-containing protein |
| Os.9991.1.S1_at | 0.0467 | 2.294 | Os07g0258100 | AK064037 | Emb1030 (embryo defective 1030) |
| OsAffx.23277.2.S1_x_at | 0.0361 | 2.305 | Os01g0272800 | NM_001049246 | Expressed protein |
| OsAffx.11330.1.S1_at | 0.0371 | 2.309 | --- | NM_193227.1 | Expressed protein |
| Os.52507.1.S1_at | 0.0356 | 2.321 | Os01g0618400 | AK067570 | DEAD box RNA helicase (RH26); Identical to DEAD-box ATP-dependent RNA helicase 26 (RH26) |
| Os.46500.2.S1_at | 0.0386 | 2.321 | Os05g0269800 | NM_001061590 | Transposon protein, putative, |
| Os.37247.1.S1_at | 0.0361 | 2.322 | Os11g0116400 | AK059833 | Elongation factor P (EF-P) family protein; similar to elongation factor P (EF-P) family protein [Arabidopsis thaliana] |
| OsAffx.24745.1.S1_at | 0.0437 | 2.331 | --- | LOC_Os02g45940.1 | Histone 4 |
| Os.25613.1.A1_s_at | 0.0347 | 2.336 | Os02g0148000 | AK120401 | Transcription regulator responsible for specific upregulation of the translocon genes attoc33 and attoc75 in leaves. Involved in protein import into chloroplast. |
| Os.27782.1.S1_at | 0.04 | 2.362 | Os03g0736400 | AK105812 | Similar to Conserved hypothetical protein 95 [Medicago truncatula] |
| Os.37673.1.S1_at | 0.0344 | 2.365 | --- | AK058411 | Expressed protein |
| Os.51299.1.S1_at | 0.0361 | 2.368 | Os05g0464300 | AK062470 | Expressed protein |
| Os.27638.1.S1_at | 0.0436 | 2.423 | Os05g0555600 | AK070485 | NADH-dependent glutamate synthase |
| Os.26862.1.S1_s_at | 0.0361 | 2.43 | Os11g0140700 | CB650128 | Binding / catalytic; similar to unknown [Nuphar advena] (GB:ABY60455.1); contains interpro domain NAD(P)-binding; (interpro:IPR016040); contains interpro domain Saccharopine dehydrogenase (interpro:IPR005097) |
| Os.51391.1.S1_at | 0.0361 | 2.435 | Os02g0773500 | AK062694 | Expressed protein |
| Os.40288.1.S1_x_at | 0.0435 | 2.442 | --- | AK242881 | Expressed protein |
| Os.35808.1.S1_at | 0.0437 | 2.465 | Os01g0606900 | AK065697 | DNAJ heat shock N-terminal domain-containing protein; similar to DNAJ heat shock N-terminal domain-containing protein |
| Os.19872.1.S1_at | 0.0471 | 2.467 | Os02g0596000 | AK072766 | Similar to rhodanese-like domain-containing protein [Arabidopsis thaliana] (TAIR:AT3G08920.1) |
| Os.38278.1.S1_at | 0.0457 | 2.489 | Os03g0164400 | AK070845 | FRS12 (FAR1-RELATED SEQUENCE 12); zinc ion binding; similar to FRS7 (FAR1-RELATED SEQUENCE 7) |
| Os.11874.1.S1_s_at | 0.0436 | 2.506 | Os07g0287100 | AK066004 | Binding; similar to unknown [Populus trichocarpa] (GB:ABK95143.1); contains interpro domain Tetratricopeptide-like helical |
| Os.54093.1.S1_at | 0.0378 | 2.533 | Os04g0566600 | AK101849 | DNA binding / transcription factor; Identical to Putative Myb family transcription factor At1g14600 [Arabidopsis Thaliana] |
| Os.9859.2.S1_at | 0.0361 | 2.586 | Os01g0188900 | AK066235 | Ankyrin repeat family protein; similar to stress-inducible protein, |
| Os.27221.1.A1_at | 0.0361 | 2.613 | Os03g0856100 | AK067137 | Pentatricopeptide (PPR) repeat-containing protein |
| Os.10293.1.S1_at | 0.0492 | 2.625 | Os07g0617000 | AK109360 | Encodes a member of the ERF (ethylene response factor) subfamily B-2 of ERF/AP2 transcription factor family (RAP2.2). |
| Os.8897.1.S1_at | 0.0356 | 2.627 | --- | AK107786 | Expressed protein |
| Os.12629.1.S2_at | 0.0361 | 2.64 | --- | AK068661 | Expressed protein |
| Os.7308.2.S1_at | 0.0361 | 2.677 | Os03g0425000 | AK120385 | Protein contains putative RNA binding domain. Expressed in response to Pseudomonas syringae infection. Resistance requires silencing of atrap suggesting it functions as a negative regulator of plant disease resistance. |
| Os.9300.1.S1_at | 0.0361 | 2.69 | Os05g0360400 | AK106046 | Encodes a RING finger domain protein with E3 ligase activity that is localized to the lipid rafts of the plasma membrane. Expression is increased in response to fungal pathogen. |
| Os.17075.1.S1_at | 0.049 | 2.691 | Os03g0769600 | AK100054 | Cytochrome c biogenesis protein family |
| OsAffx.3461.1.S1_at | 0.0488 | 2.692 | Os03g0602600 | NM_001057160 | Putative fructokinase |
| Os.4156.1.S1_at | 0.0456 | 2.705 | Os06g0486800 | AB019533 | Nad-dependent formate dehydrogenase |
| OsAffx.12022.1.S1_s_at | 0.0347 | 2.708 | --- | LOC_Os02g13600.1 | Expressed protein |
| Os.19456.1.S1_at | 0.0361 | 2.71 | Os02g0224900 | AK101829 | Peroxisomal membrane protein (PMP36); similar to ATFOLT1 (ARABIDOPSIS THALIANA FOLATE TRANSPORTER 1), |
| Os.51357.1.S1_at | 0.0378 | 2.712 | Os07g0641400 | AK062634 | Expressed protein |
| Os.47722.1.S1_at | 0.0425 | 2.73 | Os03g0439800 | AK058503 | Integral membrane protein |
| Os.5363.1.S1_at | 0.0463 | 2.743 | --- | AK241402 | Putative cadmium-induced protein |
| Os.15428.1.S1_at | 0.0359 | 2.772 | Os01g0702000 | AK065414 | Wound induced gene |
| Os.52476.1.S2_at | 0.0401 | 2.787 | Os02g0120000 | AK067383 | Inactivation of the chloroplast ATP synthase gamma subunit results in high non-photochemical fluorescence quenching and altered nuclear gene expression in Arabidopsis thaliana |
| Os.50255.1.S1_at | 0.0465 | 2.796 | Os06g0360600 | AK120346 | Expressed protein |
| Os.52638.1.S1_at | 0.0467 | 2.802 | Os02g0827600 | AK068455 | Similar to oxidoreductase/ transition metal ion binding [Arabidopsis thaliana] (TAIR:AT4G29400.1) |
| Os.25324.1.A1_at | 0.0426 | 2.805 | Os08g0224300 | CA760624 | Proton extrusion protein-related; similar to unnamed protein product [Vitis vinifera] (GB:CAO66548.1); contains interpro domain cema; (interpro:IPR004282) |
| Os.27165.1.A1_at | 0.0361 | 2.86 | Os09g0506300 | NM_001070143 | Expressed protein |
| Os.40210.1.S1_at | 0.049 | 2.867 | Os07g0222900 | CB645570 | Expressed protein |
| Os.56168.1.S1_at | 0.0361 | 2.876 | Os05g0482400 | AK109526 | Cytochrome P450 72A1, putative, expressed (EUI) |
| Os.13862.1.S1_at | 0.0417 | 2.968 | --- | AK070747 | CPZ [Arabidopsis thaliana] sp|Q8L633|RNZC_ARATH recname |
| Os.35524.1.S1_at | 0.0361 | 3.009 | Os01g0871800 | AK064787 | Expressed protein |
| Os.47955.2.S1_x_at | 0.0371 | 3.026 | Os12g0119000 | AK061990 | Expressed protein |
| Os.27765.1.S1_at | 0.0361 | 3.056 | Os06g0714800 | AK059793 | Senescence-associated protein-related; similar to senescence-associated protein-related [Arabidopsis thaliana] (TAIR:AT4G17670.1) |
| Os.50256.1.S1_at | 0.0485 | 3.064 | --- | AK120357 | Expressed protein |
| Os.18008.1.S1_at | 0.0414 | 3.068 | Os01g0127400 | CB653148 | Myb-like gene that may act as a negative regulator of trichome branching |
| Os.49191.1.S1_at | 0.0361 | 3.111 | Os09g0382400 | AK062891 | Expressed protein |
| Os.52275.1.S1_x_at | 0.0492 | 3.143 | Os12g0614900 | AK066039 | Encodes a wall-associated kinase |
| Os.47949.1.S1_a_at | 0.0455 | 3.149 | Os05g0592300 | AK068520 | Expressed protein |
| OsAffx.3569.1.S1_x_at | 0.0421 | 3.188 | Os03g0718800 | AK288124 | Expressed protein |
| Os.28976.1.S1_at | 0.0359 | 3.198 | Os01g0205200 | AK110817 | Pentatricopeptide (PPR) repeat-containing protein |
| Os.19052.1.S1_at | 0.0344 | 3.302 | --- | AK073452 | Expressed protein |
| Os.52588.1.S1_at | 0.0345 | 3.468 | Os07g0489800 | AK068191 | Ferredoxin-related; similar to ferredoxin-related [Arabidopsis thaliana] (TAIR:AT4G32590.1); |
| OsAffx.23061.1.S2_at | 0.0471 | 3.507 | --- | AK064564 | Expressed protein |
| OsAffx.12071.1.S1_s_at | 0.0361 | 3.543 | Os02g0266000 | AK242246 | Phosphoribosylanthranilate isomerase |
| OsAffx.30109.1.S1_at | 0.0426 | 3.56 | Os09g0489200 | NM_001070068 | Transcription factor jumonji (jmjc) domain-containing protein |
| Os.56280.1.S1_at | 0.0359 | 3.736 | Os02g0160800 | AK109752 | Wound-responsive protein, putative; similar to wound-responsive family protein [Arabidopsis thalia |
| Os.2544.1.S1_s_at | 0.0391 | 3.756 | Os10g0492600 | AB114828 | Alpha-tip, alpha-tonoplast intrinsic protein, t18k17.14, t18k17_14, tip3 |
| Os.53237.1.S1_at | 0.0361 | 3.776 | Os07g0524900 | AK071512 | Nodulin mtn21 family protein; similar to nodulin mtn21 family protein [Arabidopsis thaliana] (TAIR:AT3G53210.1); |
| Os.6042.1.S1_at | 0.0345 | 3.777 | Os07g0440100 | AU166923 | Expressed protein |
| Os.25367.1.A1_at | 0.0457 | 3.855 | --- | AK242267 | Expressed protein |
| Os.5248.1.S1_s_at | 0.0448 | 3.987 | Os04g0675400 | AK068186 | DNAJ heat shock N-terminal domain-containing protein; similar to DNAJ heat shock N-terminal domain-containing protein [Arabidopsis thaliana |
| Os.57132.1.S1_at | 0.0426 | 4.235 | Os02g0222100 | AK110984 | Arabidopsis thaliana O-acetylserine (thiol) lyase (OAS-TL) isoform oasc |
| Os.36278.1.S1_x_at | 0.0456 | 4.521 | Os03g0129400 | AK063857 | Expressed protein |
| Os.36969.1.S1_at | 0.0435 | 4.732 | Os04g0119000 | AK120328 | Expressed protein |
| Os.15247.1.S1_s_at | 0.0356 | 4.783 | Os12g0586100 | AB125310 | Snf1-related protein kinase 2-2, snf1-related protein kinase 2.2, snrk2-2, snrk2.2, spk-2-2, srk2d, t20e23.100 |
| Os.52655.1.S1_at | 0.0435 | 4.94 | Os02g0223300 | AK068568 | Nucleotidyltransferase family protein; similar to npap (NUCLEAR POLY(A) POLYMERASE) [Arabidopsis thaliana] |
| Os.19061.1.S1_s_at | 0.0457 | 4.966 | Os06g0474800 | AK099499 | DNAJ heat shock N-terminal domain-containing protein; similar to DNAJ heat shock N-terminal domain-containing protein [Arabidopsis thaliana |
| Os.27345.1.S1_at | 0.0485 | 5.023 | Os04g0243400 | AK070633 | Expressed protein |
| Os.22703.1.S1_at | 0.0389 | 5.066 | Os07g0618700 | AK066033 | Mbd2. Similar to unknown protein [arabidopsis thaliana] (tair:at4g33980.1); similar to hypothetical protein mtrdraft_ac151424g24v1 [medicago truncatula] (gb:abe88920.1) |
| Os.487.1.S1_at | 0.0437 | 5.104 | Os01g0257300 | AK070626 | Encodes a small protein with unknown function and is similar to flower promoting factor 1. This gene is not expressed in apical meristem after floral induction but is expressed in roots, flowers, and in low abundance, leaves |
| Os.48053.1.A1_at | 0.0371 | 5.294 | Os07g0142100 | AA753122 | Expressed protein |
| Os.8480.1.S1_at | 0.0361 | 5.528 | Os05g0369900 | AK064110 | Expressed protein |
| Os.313.1.S1_a_at | 0.0398 | 5.629 | Os02g0730000 | AB030939 | Mitochondrial aldehyde dehydrogenase ALDH2a /FL |
| Os.48057.1.S1_at | 0.0436 | 5.759 | Os03g0237100 | AK073738 | Aldo/keto reductase, putative; similar to aldo/keto reductase, putative [Arabidopsis thaliana] |
| Os.49818.1.S1_s_at | 0.0378 | 5.89 | Os07g0537500 | AK111734 | Protein kinase family protein; Identical to Cysteine-rich receptor-like protein kinase 8 precursor (CRK8) [Arabidopsis Thaliana] (GB:O65468); |
| OsAffx.26649.1.S1_x_at | 0.0437 | 6.024 | --- | AK068186 | Zinc finger (C3HC4-type RING finger) family protein |
| Os.50611.1.S1_x_at | 0.0344 | 6.259 | Os02g0221900 | AK122077 | Cytochrome P450 monooxygenase CYP711A, putative, expressed |
| Os.18633.1.S1_at | 0.0378 | 6.365 | Os04g0517500 | AK066885 | Calcium-dependent protein kinase, putative, expressed |
| OsAffx.18687.1.S1_x_at | 0.0436 | 6.397 | --- | AK102457 | Major Facilitator Superfamily, putative |
| Os.6315.1.S1_at | 0.0361 | 6.49 | --- | AK101374 | Expressed protein |
| OsAffx.31535.1.S1_at | 0.0386 | 8.361 | Os11g0103400 | NM_001072055 | IBR domain containing protein |
| Os.34992.2.S1_x_at | 0.0361 | 8.998 | --- | AK069394 | Putative cytochrome P450, 5'-partial |
| Os.53493.1.S1_at | 0.0361 | 9.339 | Os05g0563300 | AK072939 |  |
| Os.27900.1.A1_at | 0.0378 | 9.898 | --- | AK060432 | Expressed protein |
| Os.53657.1.S1_at | 0.0471 | 10.22 | Os08g0292000 | AK100441 | Encodes a homeobox-leucine zipper family protein belonging to the HD-ZIP IV family |
| Os.10251.1.S1_at | 0.0425 | 10.95 | Os10g0472900 | AK120294 | Putative fatty acid elongase 3-ketoacyl-coa synthase |
| Os.39255.1.A1_at | 0.0488 | 12.11 | --- | AK241665 | Expressed protein |
| OsAffx.28294.2.S1_at | 0.0361 | 12.58 | --- | LOC_Os07g05840.1 | Expressed protein |
| Os.50395.1.S1_at | 0.0384 | 12.89 | Os12g0493900 | AK121028 | RNA binding [Arabidopsis thaliana] (TAIR:AT1G22240.1); |
| Os.53610.1.S1_at | 0.0455 | 13.37 | Os02g0262800 | AK073576 | Similar to resistance complex protein I2C-2 GB:AAB63275 (Lycopersicon esculentum); |
| Os.10018.1.S1_at | 0.0435 | 14.13 | Os02g0650300 | AK063464 | Transposon also (transporter) |
| Os.20614.4.S1_x_at | 0.0361 | 14.64 | Os10g0567900 | NM_001071969 | Putative transposase |
| Os.29043.1.S1_at | 0.0425 | 15.38 | Os01g0692400 | AK110723 | Expressed protein |
| OsAffx.31583.1.S1_at | 0.0492 | 15.63 | --- | LOC_Os12g04790.1 | Expressed protein |
| Os.54195.1.S1_at | 0.0435 | 17.65 | Os11g0151500 | AK102457 | Tetracycline transporter activity |
| Os.47934.1.S1_x_at | 0.0446 | 24.38 | Os11g0103000 | AK072365 | RNA binding protein involved in the processing of chloroplast psbb-psbt-psbh-petb-petd transcript unit. |
| OsAffx.28454.1.S1_s_at | 0.0436 | 26.17 | --- | LOC_Os07g15130.1 | Expressed protein |
| Os.47934.1.S1_at | 0.0361 | 30.73 | Os11g0103000 | AK072365 | RNA binding protein involved in the processing of chloroplast psbb-psbt-psbh-petb-petd transcript unit. |
| OsAffx.22999.1.S1_at | 0.0344 | 36.01 | Os07g0511400 | AK108619 | Expressed protein |
| Os.18707.1.S1_at | 0.0344 | 40.4 | Os11g0134900 | AK069533 | Carbohydrate transmembrane transporter activity, sugar:hydrogen symporter activity |
| OsAffx.11849.1.S1_s_at | 0.0456 | 46.53 | --- | AK241665 | Expressed protein |
| OsAffx.28294.2.S1_x_at | 0.0365 | 53.71 | --- | LOC_Os07g05840.1 | Expressed protein |
| Os.54747.1.S1_at | 0.0344 | 62.76 | --- | AK106655 | Expressed protein |
| Os.18676.1.S1_at | 0.0436 | 67.07 | Os02g0264700 | AK101666 | Expressed protein |
| Os.54747.1.S2_at | 0.0361 | 69.22 | --- | AK109584 | Expressed protein |
| OsAffx.27273.1.S1_at | 0.0361 | 70.56 | Os05g0491700 | NM_001062446 | Hypothetical protein [Oryza sativa (japonica cultivar-group)] |
| OsAffx.4161.1.S1_at | 0.0361 | 79.84 | Os04g0620000 | AK240686 | ABC transporter transmembrane region, putative |
| Os.46576.1.S1_at | 0.0361 | 83.49 | Os10g0504900 | AK063117 | Putative lipid transfer protein |
| Os.17918.1.S1_at | 0.0435 | 95.65 | Os03g0277600 | AK061425 | Integral membrane protein |
| Os.51290.1.S1_at | 0.0347 | 122 | --- | AK062437 | Expressed protein |
| Os.10374.2.S1_at | 0.0494 | 343.6 | Os12g0118400 | AK111804 | Expressed protein |
