# Supplementary material for: The APETALA-2-Like Transcription Factor OsAP2-39 Controls Key Interactions between Abscisic Acid and Gibberellin in Rice
Source: PLoS Genet. 2010 Sep 9;6(9):e1001098. doi: 10.1371/journal.pgen.1001098 (PMC2936520; doi:10.1371/journal.pgen.1001098)
Supplement: Table S2 — Primers used in the qRT-PCR. (0.05 MB DOC) [file pgen.1001098.s007.doc]

**Supplemental Table 2. Primers used in the QRT-PCR.**

| **Gene** | **Accession Number** | **Primer Name** | **Primer Sequence (5'-3')** |
| --- | --- | --- | --- |
| *OsNUE39* | Os04g0610400 | Ap2ReS1F | TCGTCAGCGTCAAGAAGGAGG |
|  |  | Ap2ReS1R | TTCACCACGGATGACGACGAG |
| *EUI* | Os05g0482400 | Real2EU1F | ACAAGTGGAGGAAAGCCTACGG |
|  |  | Real2EU1R | AAGAGTGGCTCCTGGCCTTTCT |
| *GID1L2* | Os06g0214800 | RGI1L2F | ACGTCCAGCTCATGCCGTTC |
|  |  | RGI1L2R | CCAGTAGCGGTCGTTGAGGTC |
| *GID1L2B?* | Os07g0162900 | RGI1L2bF | GCGTCTGGTACTACGAGAGCCTC |
|  |  | RGI1L2bR | CGCGGATCCATGCAGTAGAA |
| *GID1* | Os05g0407500 | GID1F | ACATGGGTGCTCATCTCCAA |
|  |  | GID1R | CGTCAGGAACTCAAGGATGG |
| *GA-2 Oxidase* | Os04g0522500 | RGAOXIdaseF | TCGAAACCAAGGAGAAGGCC |
|  |  | RGAOXIdaseR | TGCATCACGCCCCTTAAGG |
| *SD1* | Os01g0883800 | SD1F | GCCAATGGGGAGGGTGTAC |
|  |  | SD1R | CTGGAGGAGGATGGTGAGGG |
| *OsCED1* | Os03g0645900 | OSCED1F | CGACGTGATCAAGAAGCCGTA |
|  |  | OSCED1R | TCTCCTGGAGCTTGAACACCA |
| *OsCED3* | Os07g0154100 | OSCED3F | CGCTCAGCTACAATGTCGTGTC |
|  |  | OSCED3R | ATAGTTCTCGGTGACGGCGAA |
| *OsZEP1* | Os04g0448900 | OSZEP1F | TCCGGCTCCTGGTACATCAA |
|  |  | OSZEP1R | CCAACCGCACGAGCAAGA |
| *OsMax-1* | Os02g0221900 | ROSmax1F | GGCTCTGCTTGCTGTGTTTCTG |
|  |  | ROSmax1R | CCCATGTGAAACCTGAAGATCG |
| *MOC1* | Os06g0610300 | MOC1F | CAGCTAGACCAGGTGTGGGG |
|  |  | MOC1R | CTACACACGCAATGCTTTAAATGG |
| *Auxin response factor* | Os01g0753500 | Os01g0753500F | TGGTGGTTCAATCTCTGTTGCT |
|  |  | Os01g0753500R | CGACCATTTCCATACAGAGCTG |
| *SAUR_B* | Os02g0769100 | Os02g0769100F | CGTAGCTTGATTGCACTTGCG |
|  |  | Os02g0769100R | AAGCACCAACCACCGAAACAG |
| *Auxin-responsive* | Os10g0510500 | Os10g0510500F | TCCCTTTAACCCCATCAAACG |
|  |  | Os10g0510500R | CCGTGCATCTCGCAACAAT |
| *Actin 2* | Os10g0510000 | 36650F | TCTTACGGAGGCTCCACTTAAC |
|  |  | 36650R | TCCACTAGCATAGAGGGAAAGC |
